# Classification errors distort findings in automated speech processing: Examples and solutions from child-development research

**DOI:** 10.3758/s13428-026-03029-6

**Published:** 2026-05-18

**Authors:** Lucas Gautheron, Evan Kidd, Anton Malko, Marvin Lavechin, Alejandrina Cristia

**Affiliations:** 1 Evolution, Science and Society, University of Missouri, Columbia, MO US; 2 University of Wuppertal, Wuppertal, Germany; 3 Laboratoire de Sciences Cognitives et Psycholinguistique, Département d’études cognitives, ENS, EHESS, CNRS, PSL University, Paris, France; 4 School of Literature, Languages and Linguistics, Australian National University, Canberra, Australia; 5 Laboratoire d’Informatique et Systèmes, Université Aix-Marseille, CNRS, Marseille, France

**Keywords:** Language acquisition, Long-Form recordings, Speech processing, Classification bias, Event detection, Latent variable modeling

## Abstract

With the advent of wearable recorders, scientists are increasingly turning to automated methods of analysis of audio and video data in order to measure children’s experience, behavior, and outcomes, with a sizable literature employing long-form audio-recordings to study language acquisition. While numerous articles report on the accuracy and reliability of the most popular automated classifiers, less has been written on the downstream effects of classification errors on measurements and statistical inferences (e.g., the estimate of correlations and effect sizes in regressions). This paper’s main contributions are drawing attention to downstream effects of confusion errors, and providing an approach to measure and potentially recover from these errors. Specifically, we use a Bayesian approach to study the effects of algorithmic errors on key scientific questions, including the effect of siblings on children’s language experience and the association between children’s production and their input. By fitting a joint model of speech behavior and algorithm behavior on real and simulated data, we show that classification errors can significantly distort estimates for both the most commonly used Language ENvironment Analysis (LENA™), and a slightly more accurate open-source alternative (the Voice Type Classifier from the ACLEW system). We further show that a Bayesian calibration approach for recovering unbiased estimates of effect sizes can be effective and insightful, but does not provide a foolproof solution.

**Supplementary Information:**

The online version contains supplementary material available at 10.3758/s13428-026-03029-6.

## Introduction

Children’s behavior and their environments are increasingly described through automated analysis of data collected from wearables. Pioneers in such techniques, researchers working on language acquisition have shown the promise of using automated classifiers to analyze long-form audio recordings, thus enabling the processing of naturalistic data at unprecedented scale (Bergelson et al., 2023). A common application in this context is the automatic segmentation and classification of speech into voice categories for measuring speech afforded to and produced by infants. A growing number of studies document and discuss the accuracy of these automated classifiers (such as Language ENvironment Analysis (LENA™), by comparing human and machine annotations of the same audio clips (Lavechin, Hamrick, Kelleher, & Seidl, 2025; Xu, Yapanel, & Gray, 2009). Concern has been raised about unexpected cases of low recall and precision (e.g., a precision of 27% for the recognition of the child wearing the device in Gilkerson et al., 2015), as well as trends for confusion across speaker types (Lehet, Arjmandi, Houston, & Dilley, 2021). To our knowledge, less attention has been paid to how classification errors propagate through subsequent analyses. This paper examines a critical question: To what extent do errors in automated speech processing systems like LENA™ affect downstream measurements and scientific conclusions? Specifically, we investigate how speaker tagging misclassifications (e.g., confusing child speech with adult speech) impact measurements of children’s linguistic input and production, as well as statistical estimates (e.g., effect sizes) derived from these measurements. To study this, we introduce a Bayesian approach that simultaneously models speech behavior as well as algorithm behavior. This joint model allows us to shed light on the profound and downstream consequences of algorithmic errors. Additionally, we take this investigation a step further by using the joint model to recover unbiased measurements that account for algorithmic errors. The methodological insights gained here may also apply to signals captured by other wearable technologies (e.g., video; Long et al., 2024), and generally, to any case where event detection is performed in conjunction with classification using machine learning algorithms with non-zero error rates. However, both for clarity and to better inform one specific community, we focus on the case of voice type classifiers.

**Fig. 1:**
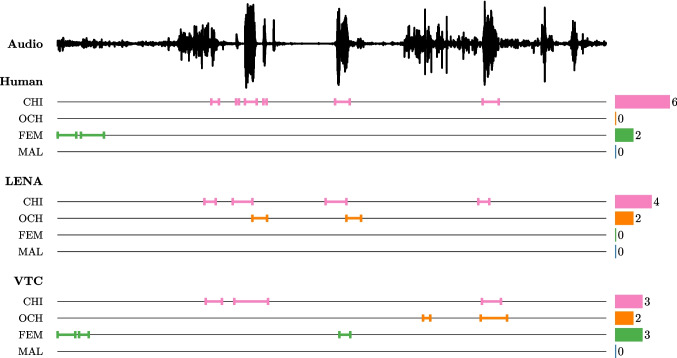
A 30-s sample of a daylong recording annotated by a human expert and two algorithms: LENA™, and VTC. CHI refers to the child wearing the recording device; OCH refers to other children; FEM and MAL refer to female and male adults. A segment of speech is referred to as a “vocalization” (for instance, the expert found two female adult vocalizations in this portion of audio, but LENA™ found none). Vocalization counts are shown to the right

### The case of voice type classifiers when describing early language acquisition

Day-long audio recordings of children’s language experience collected with wearable devices have become widespread thanks to their richness and ecological validity. The adoption of this methodology has been facilitated by LENA™, a user-friendly commercial solution simplifying data collection and analysis. LENA™ provides both the recording device and the software for automatically annotating the audio. The latter is important, given that this technique produces a large amount of data (up to 16–24 hours of audio per session in the case of LENA™, to be multiplied by the number of children and the number of sessions per child), which would be impossible to analyze entirely by hand. Among other things, LENA™ includes a diarization algorithm that detects speech (*event detection*) and attributes it to one of four different types of speaker (*classification*): the child wearing the recording (CHI), another child – e.g., a sibling – (OCH), female adult (FEM), and male adult (MAL).^1^ With additional processing steps that build on this essential diarization algorithm, LENA™ provides a number of metrics, including the child vocalization count (CVC; i.e., the number of speech-like segments attributed to the key child). Researchers were quick to consider potential errors in the algorithm. Processing audio data collected directly from children in noisy real-life conditions is challenging, and the metrics returned by LENA™ (or any other classifier) are far from perfect. Yet, LENA™ is generally considered to have been sufficiently validated, with a meta-analysis (Cristia, Bulgarelli, & Bergelson, 2020) finding that the correlation between human and LENA™ CVC in the same audio clips averaged Pearson $R^2=0.77$ $(N=5)$. Moreover, another meta-analysis found that CVC correlated with concurrent and/or longitudinal standardized measures of language (Pearson $R^2=0.33$, $N=10$; Wang, Williams, Dilley, & Houston, 2020).

While validation studies are undeniably important, we believe that computing performance metrics alone are insufficient and that insufficient consideration has been given to how algorithms’ errors may impact conclusions. We illustrate this on the widely used vocalization count measure. Figure 1 shows the segmentation into speaker categories of a 30-s audio clip made by a human expert, the proprietary LENA™ algorithm, and its open-source alternative, Voice Type Classifier (VTC) (Gilkerson et al., 2015; Lavechin, Bousbib, Bredin, Dupoux, & Cristia, 2020). Errors in the automated annotations result in erroneous vocalization counts. For instance, in this example, both LENA™ and VTC incorrectly report two vocalizations from siblings.

**Fig. 2:**
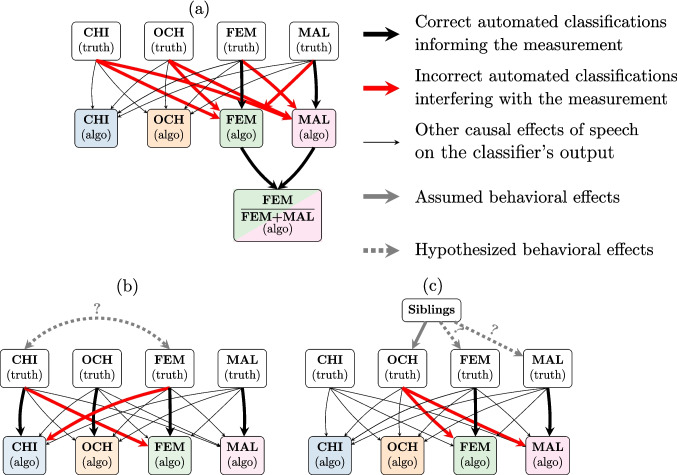
The quantity of speech attributed to each speaker (“CHI”, “OCH”, “FEM”, “MAL”, i.e. the key child, other children, female adults, and male adults) in each recording by an algorithm only indirectly reflects the true quantities. In reality, speaker classification errors can distort measurements and create spurious correlations in the quantities of speech attributed to each speaker. (**a**) Measurements of speech quantities. The nature of the input to children may be misrepresented as a result of classification errors. For instance, the proportion of female adult speech can be distorted due to incorrect inferences about the speaker’s type and gender. (**b**) Associations between speakers. An increase in female adult speech may trigger an increase in detected amounts of both female adult (black arrow) *and* child speech (red arrow), and vice-versa, creating the appearance of an association between the two speakers. (**c**) Effect of independent variables on speech quantities. Spurious associations can also affect inferences about the effect of independent variables on speech behavior. For example, we might draw incorrect conclusions about the existence and direction of an effect of siblings on the quantity of speech received from adults (dashed lines) if speech from siblings is incorrectly classified as adult speech (then, children with siblings might falsely appear to receive more input from adults)

In the literature, these measurement errors are generally considered as random noise and left untreated. This ignores the fact that the quantity of speech attributed to a given voice type can be systematically affected by the quantity of speech from others (Fig. 2). For instance, the proportion of female adult speech can be overestimated due to children being confused with a female adult, or distorted due to male and female adults being confused with one another (Fig. 2a). Specifically, in our data, we will show that VTC and LENA™ systematically distort the proportion of female adult speech. In addition, since these algorithms have different error-patterns, LENA™ generally reports higher proportions of female speech than VTC^2^; this means that comparing and aggregating findings obtained with different algorithms is highly challenging.

Additionally, and perhaps more surprisingly, classification errors can also affect estimates of the association strength in the quantities of vocalizations attributed to different voice types, and even with other variables. To see why, it is useful to frame the problem in terms of causal diagrams (specifically Directed Acyclic Graphs, or DAGs), in which directed arrows represent causal relationships between variables. Causal paths introduce correlations between variables: for example, in the causal chain ‘$\text {exposure } [e]\rightarrow \text {mediator } [m]\rightarrow \text {outcome } [o]$’ (describing a model where an exposure causes a mediator which itself causes an outcome), *e*, *m* and *o* will all appear to be correlated. In this framework, we may distinguish relevant and biasing causal paths: “[relevant] causal paths start at the exposure [*e*], contain only arrows pointing away from the exposure [*e*], and end at the outcome [*o*]” — that is, “they have the form $e \rightarrow x_1 \rightarrow \dots \rightarrow x_k \rightarrow o$.”. By contrast, “biasing paths are all other paths from exposure to outcome, [e.g.] $e \leftarrow x_1 \rightarrow \dots \rightarrow x_k \rightarrow o$” (Textor, 2015). In the latter case, $x_1$ is typically called a “*confounder*”. From this perspective, classification errors open “biasing paths” that can create completely spurious correlations between variables in sometimes unpredictable ways. This potentially leads us to over-/under-estimate the effect of an association; to believe that an association between two variables exists when it does not; or worse, to reach incorrect conclusions about the *sign* of an effect. For instance, in Fig. 2b, female adult speech incorrectly labeled as child speech may lead to spurious correlations between our measurements of adult speech (input) and child speech (output). In Fig. 2c, the effect of siblings on adult input may be similarly distorted by classification errors, which open up a completely spurious biasing path between “siblings” and female/male adult speech.

**Fig. 3:**
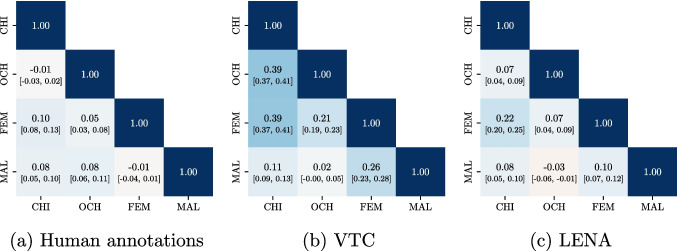
Correlations between speakers’ vocalization counts in $6638 \times 15$s audio clips according to human, VTC, and LENA™ annotations. Estimates are generally inconsistent across the three

As a brief illustration of the practical significance of these issues, we compare correlations between speaker vocalizations across 6638 $\times$ 15-s audio clips (from English-speaking corpora described in Section §“Data”) based on human, VTC, and LENA™ annotations (Fig. 3). Manual annotations reveal statistically significant but low correlations between speakers across clips (Pearson $R \le 0.10$). In contrast, VTC and LENA™ produce much larger correlations, and are inconsistent with each other. For instance, VTC reveals a medium correlation between CHI and OCH (Pearson $R=0.39$, $p<0.001$, $N=6638$), while LENA™ finds a weak correlation (Pearson $R=0.07$, $p<0.001$, $N=6638$), and manual annotations find no discernible correlation whatsoever (Pearson $R=-0.01$, $p=0.527$, $N=6638$). As we will show in §“Comparing LENA™ and VTC against manual coding”, these discrepancies are consistent with biasing paths resulting from speaker misclassification.

### Previous relevant work

Thus, while the accuracy, reliability, and validity of LENA ™ has been well documented in previous work for many languages (for meta-analyses see Cristia et al., 2021; Wang et al., 2020; see also Bastianello, Lorenzini, Nazzi, & Majorano, 2023; Bruyneel, Demurie, Boterberg, Warreyn, & Roeyers, 2021; McDonald, Kwon, Kim, Lee, & Ko, 2021; Cristia et al., 2024), the downstream effect of classification errors, for example on correlational analyses, has not been properly assessed. In the context of long-form recordings for language acquisition, the issue was briefly raised in Cristia, Gautheron, & Colleran (2023), a descriptive report on 38 children’s language input and output, and in which an attempt was made to discern whether correlations observed between speakers’ speech quantities were entirely consistent with classification errors.

In parallel, the downstream effects of errors in automated predictions (such as classification errors) have become more widely recognized in other disciplines, due to the increasing recourse to machine learning for data processing. Most prominently, Angelopoulos, Bates, Fannjiang, Jordan, and Zrnic (2023b) proposed a general approach to the issue (prediction-powered inference), which they have demonstrated on an array of datasets from different fields (biology, astrophysics, ecology, etc.). However, their strategy is currently limited to simple usages (e.g. ordinary least-squares linear regression) and lacks flexibility for complex hierarchical models such as those examined in the present paper^3^. In addition, their approach requires human labeling for a substantial amount of observations. This makes it hard to generalize to the case of voice-type classifiers, since no recording can be entirely annotated by hand (only a handful of clips at best). In a different context, TeBlunthuis, Hase, and Chan (2024) insisted that “current practices of ‘validating’ [automated classifiers] by making misclassification rates transparent via metrics such as the F1 score [...] provide little safeguard against misclassification [...]”. Independently from us and building upon prior literature on measurement errors (Carroll, Ruppert, Stefanski, & Crainiceanu, 2006, Ch. 8,15), they proposed a solution (“maximum likelihood adjustment”) conceptually similar to ours but limited to classification tasks^4^. In a nutshell, they propose averaging statistical estimates over all possible true labels, weighted by their likelihoods, based on the classifier’s predictions and possibly other covariates. Teblunthuis et al. (2024) set aside a Bayesian treatment for future work, acknowledging that “[this] may provide additional strengths in flexibility and uncertainty quantification”. Therefore, the current paper makes a distinct contribution with respect to Teblunthuis et al. (2024), through a flexible Bayesian approach and by overcoming the restriction to classification tasks. Additionally, we are motivated to make these contributions accessible to a wider readership interested in behavioral research methods.

### Present work

The present paper seeks to make two contributions: First, highlighting the many effects of confusion errors; and second, providing a first approach to recovering from these errors. We study voice type classification errors by two popular algorithms: LENA™, the historically dominant solution in the field, and VTC, a state-of-the-art alternative gaining traction (Laudańska et al., 2025). Both were applied to the very same longitudinal audio data (1 401 recordings of $\sim$8 hours each from 217 children across six corpora; see Section §“Data”), allowing us to document the effects of classification errors on vocalization quantities, but also on a variety of theorized language acquisition factors. To better inform our readership, we assess the consequences of classification errors on a variety of research problems (Fig. 2): (a) direct measurement of speech quantities (in our example, the proportion of female adult input); (b) measurements of associations between speech quantities (the short and long term effects of input on output); and (c) measurements of the effect of an independent variable on speech quantities (respectively, the effect of the child’s age on its vocal production, and the effect of sibling number on the child’s experience). This calls for a multi-level hierarchical model of speech behavior which allows us to exploit the longitudinal aspects of included data, assuming that many researchers will be interested in both developmental effects (e.g., does more adult input lead children to vocalize more over time?) as well as household effects (does the presence of siblings lead to changes in adult vocalization counts?).

At the heart of our approach is the requirement that a theoretically driven model of speech behavior be complemented by a model of how the algorithm behaves. We inform our algorithm behavior model with $\sim$28h of human-annotated data, which allows us to model the relationship between the *true* but unobserved vocalization counts of each different talker type (male adults, female adults, other children, and the key child) and the vocalization counts provided by the diarization algorithms in the same data. In other words, even though there is no data directly showing how counts by talker type are misestimated at the recording level, our model can learn from errors occurring in the shorter time-scales that have been human-annotated.

As a brief summary, we find that classification errors can significantly distort estimates of speech quantities and effect sizes in downstream analyses, sometimes even leading to incorrect conclusions about the existence, size, and (possibly) the direction of a correlation/effect. Furthermore, the impact of classification errors varies depending on the precise effects being estimated. For instance, while the effect of having siblings on the quantity of speech from each speaker is significantly distorted by measurement errors, distortions are smaller for developmental effects unfolding throughout child development, such as the long-term effect of input on output.

In addition to being the first to highlight these widespread and complicated effects of confusion error on potential scientific inferences, this paper sought to make a second contribution by assessing whether the proposed joint model approach may also help recover unbiased estimates. We provide a range of evidence suggesting that joint modeling does improve things. For instance, we examined the extent to which LENA™ and VTC yielded the same results: if our joint model approach unbias the estimates, then the two algorithms’ outputs should come closer to the underlying truth. We indeed found that our approach improves the agreement between the two algorithms in all cases except one. While this is encouraging, we believe that the resulting Bayesian calibration model for producing unbiased estimates of correlations and effect sizes using algorithmic vocalization data may be most useful to technically proficient readers. We also provide a Python package that enables scientists to simulate the impact of classification errors on their own analyses (Gautheron, 2025), which may be easier to adopt while still requiring familiarity with scripting.

#### Outline of our methodological approach

The following is a high-level description of our methodological approach, with details being provided in the Methods section. We start by specifying a model of speech behavior that embeds our theoretical assumptions and/or the hypotheses we evaluate. Each researcher may posit a different model of speech behavior, depending on their own research questions. For the purposes of this paper, our model of speech behavior specifies that, at the recording level, children’s input composition may vary as a function of how many siblings the child has; and the key child’s output may vary as a function of their age, input, and random individual variation. This model of speech behavior is not a main contribution of this paper, but rather a necessary component of our approach, allowing us to illustrate the downstream consequences of classification errors on reasonably motivated analyses.

Next, we embed this model of speech behavior into a larger model that also accounts for the fact that we do not observe children’s “REAL” number of adult vocalizations, but instead estimate them using an algorithm. To this end, we include the effect of the algorithm in the data-generating process, treating the true vocalization counts as latent (i.e., unobserved) variables. Fortunately for the current research, several datasets have been partially annotated by humans, which means we have reasonably accurate gold-standard estimates of how many vocalizations were produced by the key child, other children, and male and female adults, for very small extracts from the long-form recordings. For this initial foray in studying how algorithmic errors may affect scientific conclusions, we assume that the most relevant features of the algorithm relate to the model’s tendency to miss vocalizations, assign them to the wrong speakers, as well as incorrectly break up or lump vocalizations.

Using a Bayesian approach, our task then becomes estimating each parameter in the joint model, using audio clips with both algorithmic and human annotations to inform the model’s algorithm, and using recording-level automatic annotations to inform parameters related to our model of speech behavior. We note that this does not allow us to “correct” the individual classification decisions made by diarization algorithms or the resulting segmentation files (e.g., the .its returned by LENA™). Calibration is intended to improve, at the recording level, estimates of aggregate quantities of vocalizations and their relationships, within the context of a specified model of speech behavior.^5^ This approach is highly flexible, since it decouples the statistical model of speech behavior from the model of the algorithm’s behavior: both can be refined in parallel as two distinct modules, regardless of their respective complexity. Finally, as an alternative to Bayesian calibration, the joint model can be used in simulations for traditional null hypothesis testing.

The technical details are elaborated in Section (§“Method”). Readers mostly interested in our findings and their implications may jump to the Results section directly (§“Results”).

## Method

In this Section, we introduce the Bayesian calibration approach (§“Bayesian calibration of algorithmic vocalization counts”), a simulation-based alternative to Bayesian calibration (§“An alternative to calibration: simulations for sensitivity analysis and null-hypothesis testing”), and finally, the data used in the present study (§“Data”).

### Bayesian calibration of algorithmic vocalization counts

#### Principle

Traditionally, scientists directly fit a model of speech behavior against automated measurements of speech data ($D_{\text {meas}}$), producing estimates and/or posterior distributions for parameters of interest entering the model (e.g., they estimate $P(\theta |D_{\text {meas}})$, where $\theta$ may be the strength of the effect of age on children’s output, or the effect of siblings on the quantity of input, etc.). However, as we explained above, when $D_{\text {meas}}$ is contaminated with classification errors (as is the case for vocalization counts derived from diarization algorithms), treating it as truth can lead to incorrect conclusions. We therefore propose a Bayesian calibration approach for learning from observations affected by classification errors. As we shall see below, a Bayesian framework offers a natural response to this challenge by simultaneously leveraging prior theoretical knowledge, manual annotations, and automated annotations into a unified approach.

Bayesian calibration combines the assumed model of speech behavior with a model of the algorithm’s behavior into a single larger model. Under the hood, this approach infers the probability distribution of the unobserved *true* amounts of vocalizations of each speaker ($\hat{D}$) given the algorithm output ($D_{\text {meas}}$), and plug those estimates into the model of behavior rather than $D_{\text {meas}}$. If $\theta$ are the parameters of interest of the model, $D_{\text {meas}}$ are the data produced by the algorithm, $\hat{D}$ are the true but unobserved quantities, and $\nu$ are nuisance parameters, then the posterior distribution of $\theta$ (the parameter(s) of interest) given the observed data $D_{\text {meas}}$ is:1The posterior distribution is factored into two terms. The first term encodes the assumed model of speech behavior. The second term encodes the algorithm’s behavior, expressed as the probability that the algorithm produces quantities $D_{\text {meas}}$ (e.g., the number of vocalizations attributed to each speaker) given the true unobserved quantities $\hat{D}$ – if the algorithm was perfect, we would have $P(D_{\text {meas}}|\hat{D})=1$ if and only if $D_{\text {meas}}=\hat{D}$, and 0 otherwise). The nuisance parameters $\nu$ characterize the algorithm’s behavior (typically, the classifier’s confusion matrix), and are a priori unknown. To learn $\nu$, one must add calibration data, for which both the algorithm’s output ($D_{\text {calib}}$) and the ground truth ($\hat{D}_{\text {calib}}$) are observed. In an ideal world, every speaker in a recording would be wearing a device that detects their vocalizations – regardless of how soft or how confusable with those of others. In our real world, however, we do not have access to this ground truth but rather to human annotations (which may miss vocalizations or confuse speakers). Incorporating a consideration of human annotations as our “ground-truth” allows us to decompose the algorithm behavior as follows:2To the extent that the model of the algorithm’s behavior is valid *and* the “ground-truth” data is correct, the posterior distribution of $\theta$ retrieved by computing this integral will be unbiased. However, the uncertainty induced by the behavior of the algorithm will widen the posterior distribution of $\theta$, given that different values of $\hat{D}$ (the true quantities of speech) are compatible with the algorithm output $D_{\text {meas}}$; in other words, this approach trades bias for variance.

**Fig. 4:**
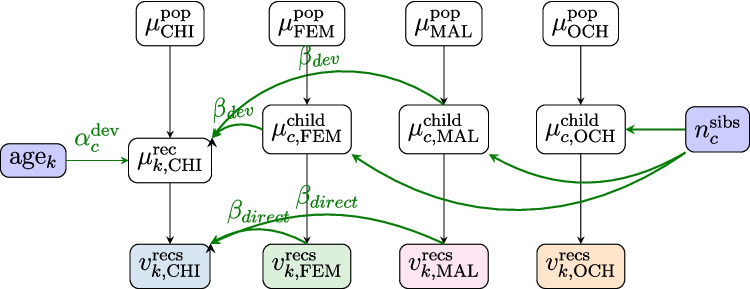
Model of speech behavior. Observed variables (vocalization counts for each speaker and recording, child age, and number of siblings) are shown in *blue*, latent variables in *white*. Indices *k* designate recordings, and *c* designates a child. $v_k^{\text {recs}}$ is the vocalization count of each speaker class in each recording. Variables $\mu$ represent the expected speech rates per speaker at each level (population, corpus, and child). $\alpha _{c}^{\text {dev}}$ is the random effect of age on the children’s output (which is assumed to be distributed around a mean value $\alpha _{\text {dev}}$). It is also assumed that the expected quantity of adult speech at the child level has a long-term effect on children’s speech ($\beta ^{\text {dev}}$), which interacts with age

#### Model of speech behavior

In Bayesian calibration, as in traditional regression analysis, we must start by specifying a model of speech behavior, encoding the relevant underlying processes (whether they operate at a cognitive or social level). We propose a multi-hierarchical model that implements several assumptions (see Fig. 4 for a visual representation, and Appendix §A.1.2 for justifications). First, the quantity of vocalizations by each speaker class (CHI, OCH, FEM, and MAL) is thought to potentially vary across children. Second, the number of siblings a child has may affect speech quantities by OCH and ADU (i.e., FEM and MAL), but not CHI directly. Third, we also assume a random child-specific effect of development on children’s speech output. Specifically, our model assumes that children’s speech quantities at birth are equivalent (i.e., individual newborns do not differ from each other), with random individual variation emerging as children age (using a Generalized Linear Model with a log link function). Finally, the model also assumes a long-term effect of adult input on children’s output (i.e., an effect of adult speech at a child-level that interacts with the children’s age). All effects of age are assumed to be log-linear, up until a threshold (24 months) after which they plateau (this threshold was validated via a change-point model). The precise model specification, including the priors on every parameter, is described in Section §A.1.1.

#### Model of the algorithm’s behavior

The calibration approach requires a model of the algorithm in terms of the probability that it outputs certain values given the unobserved true amount of vocalizations of each speaker class $i\in \{\text {CHI},\text {OCH},\text {FEM},\text {MAL}\}$, which will be further referred to as $v_i$. We assume that each of the vocalizations from each speaker *i* causes the algorithm to attribute a random amount (0, 1, 2, ...) of vocalizations to each speaker *j*, resulting in a total of $n_{ij}$ vocalizations attributed to *j* as a result of the true vocalizations from *i*. The only observable quantity is, in fact, $n_{j}=\sum _i n_{ij}$, the total amount of vocalizations attributed to each speaker *j* by the algorithm. Different assumptions could be made about how $n_{ij}$ is generated, given $v_i$, the unobserved true amount of vocalizations from each speaker *i*. For instance, one can assume a binomial process, such that the $v_i$ vocalizations are detected and attributed to *j* with probability $\lambda _{ij}$ (that is, $n_{ij}\sim \text {Binomial}(v_i,\lambda _{ij})$). However, some vocalizations are detected as multiple vocalizations (the algorithm breaks them into multiple segments), which a binomial process would fail to capture. We therefore consider a generalized Poisson distribution (from Efron, 1986), such that $n_{ij}\sim \text {DPO}(\lambda _{ij} v_i, \tau )$ with mean $\lambda _{ij}v_i$ and variance $\lambda _{ij}v_i/\tau$^6^.

In this model, $(\lambda _{ij})$ is the confusion matrix of the algorithm; the diagonal $(\lambda _{ii})$ measures the rate of true positives, and the non-diagonal elements $\lambda _{i,j\ne i}$ measure the rate of false positives due to speaker misidentification. The confusion rates $\lambda _{ij}$ are assumed to vary from one recording to another (due to unpredictable variations in recording conditions for instance), such that $(\lambda ^{k}_{ij})$ (the confusion rates for a particular recording *k*) are drawn from Gamma distributions with means $\mu _{ij}$ and shapes $\alpha _{ij} \sim \textrm{Pareto}(1,1.5)$ (truncated to values $\ge 1$^7^). $\lambda$, $\mu$, $\alpha$, and $\tau$ are nuisance parameters that can be partially learned from calibration data.

Ultimately, we implement the model in Stan (Carpenter et al., 2017), which uses a variant of Hamiltonian Monte Carlo and therefore cannot directly sample latent discrete parameters such as $n_{kij}$^8^. In the case of the calibration data, for which a “ground truth” ($v_{ki}$) is known, the solution is to marginalize over the discrete latent parameters $n_{kij}$, which comes down to computing the sum Eq. 3:

3 $$\begin{aligned} P(n_{kj}=n|v_{k1},\dots ,v_{kC}) = \sum _{0\le n_{kij}\le n} \prod _{i=1}^{C} P(n_{kij}|v_{ki})\cdot \delta (n-\sum _{i=1}^{C} n_{kij}) \end{aligned}$$

The joint knowledge of the algorithm’s vocalization counts $(n_{kj})$, and the true counts $(v_{ki})$ in manually annotated clips of audio allows us to learn the distribution of confusion rates ($\lambda$) across recordings. There is one computational caveat: the number of combinations to be summed over becomes combinatorially large for large values of $v_{i}$. Therefore, manually annotated clips are split into windows of 15 s, which keeps $v_{i}$ reasonably small with each window (originally, the duration of manually annotated clips varied between 15 s and 5 min, always being a multiple of 15 s). In fact, larger temporal windows would be less informative. However, we do not think it is wise to use even shorter time windows, since it would introduce boundary effects, and we found 15s to be a good compromise between tractability, information value, and bias.

For most of the audio, only automated annotations are available, and the true values $v_{ki}$ are unknown: they are latent parameters, and the goal is precisely to measure their distribution. To this end, we approximate $v_{ki}$ as a continuous parameter – which Stan can conveniently sample from – and assume that:

4 $$\begin{aligned} n_{kj}\sim \textrm{DPO}(\sum _{i=1}^{C} \lambda _{kij} v_{ki}, \tau ) \end{aligned}$$

where DPO designates the generalized Poisson distribution from Efron (1986).

##### Limitations

As with any model, the above simplifies reality in numerous ways. First, the effect of true vocalizations from each speaker is assumed to add linearly, which might not be a good approximation in the case of overlapping speech. This could slightly bias estimates of correlations between input and output if those were driven by dense interactions over short time scales. In fact, the model ignores that LENA™ does not handle speaker overlap. Second, confusion rates are assumed to be random and independent of variables such as the child’s age, or population-level effects (e.g., differences in environment or language). Yet, if, say, an algorithm detects vocalizations from older children more accurately, this could lead to overestimating the increase in output over time. Our approach (just like that of Teblunthuis et al., 2024) can in principle account for such biases. However, we found that we did not have access to enough human annotations to incorporate these effects directly into our full model, due to poor identification. Nevertheless, we independently assessed the effect of the child’s age and the environment (urban vs. rural) in Appendix §A.3, which did not reveal unambiguous and significant effects (see also Peurey et al., 2025). We recommend that additional work examine the potential consequences of confusion rates depending on these factors. Finally, our approach relies on the assumption that human annotations provide a reliable ground truth, but humans make mistakes. Agreement between human annotators in typical longform recordings has been reported in terms of the average F-score^9^ (across speaker types), yielding $F_1\sim 0.70$, which is far from perfect (Kunze et al., 2025). In a subset of our dataset, we evaluate the agreement between raters in terms of intra-class correlation coefficient (ICC) (by comparing vocalization counts estimates from different raters for multiple 15-second audio clips) and find $\textrm{ICC}\sim 0.7-0.9$ for humans versus humans on average, instead of 0.2-0.6 for humans versus VTC/LENA™ (Table 8, Appendix §A.5). Relatedly, our model does not accept overlapping annotations from multiple annotators. In principle, an approach combining our model with annotation models (Paun et al., 2018) such as Dawid and Skene (1979) and Albert and Dodd (2004) would have the simultaneous abilities of acknowledging human errors and aggregating redundant annotations. However, given the sparsity of human annotation available in the context of longform recordings, and the added computational complexity, such strategies may prove highly challenging for this task in practice^10^. Even if these challenges were addressed, however, humans tend to make similar errors due to biases (such as attributing loud child vocalizations to the key child regardless of their true origin), which constrains the ability of annotation models to reliably infer truth from agreement patterns.

**Fig. 5:**
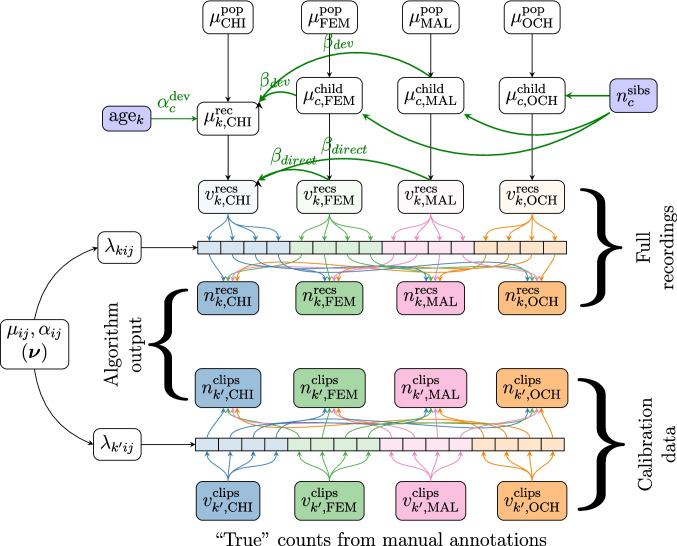
Combined model of speech behavior and of the algorithm behavior. Compared to Fig. 4, the actual quantity of vocalizations $(v^{\text {recs}})$ is treated as a latent variable. *Colored arrows* represent the effect of real vocalizations from each speaker (e.g., CHI, in  ) on the amount of vocalizations attributed to each speaker by the algorithm $(n^{\text {recs}})$. The unobserved confusion rates $\lambda _{kij}$ represent the rate at which vocalizations from a speaker *i* are detected and attributed to a speaker *j* in recording *k*. The distribution of $\lambda _{kij}$, parameterized by $\mu _{ij}$ and $\alpha _{ij}$, is learned via calibration data (for which both the true counts $n^{\text {clips}}$ and the algorithmic counts $v^{\text {clips}}$ are known)

#### The joint model

Figure 5 shows the combination of the models of speech behavior and of the algorithm. It visually emphasizes that algorithmically derived vocalization counts no longer directly inform the model of speech behavior; rather, they inform the latent true quantity of speech, which in turn informs the behavioral model. The relationship between the true and the measured vocalization counts is itself informed by the calibration data. We reiterate that this approach is highly flexible, given that the model of speech behavior and the model of the algorithm can be elaborated, reused, and improved separately as independent modules (in contrast to Angelopoulos et al., 2023b, which is practically limited to simple models, and which requires deriving specific solutions for every estimator).

##### Validation

First, we considered the algorithm model in isolation. We evaluated its capacity to predict the output of each algorithm (VTC and LENA™), given human estimates of vocalizations for each speaker in manually annotated clips (Appendix §A.2.1). We found that the prediction of our model did align with the actual automated measurements, which means that our model captures the relationship between human and automated vocalization counts. Additionally, fitting the model to simulated calibration data (for which, by design, the confusion matrix was known) confirmed that the model correctly identified the true values of the parameters (Appendix §A.2.2).

In a second step, we evaluated the calibration procedure as a whole. When fitting the model to real data, the calibration procedure generally improves the correlation between the vocalization counts estimated by LENA™ and VTC on average (see Appendix §A.2.2, Table 4). Notably, calibration increases the correlation ($R^2$) between the quantity of female adult speech measured by LENA™ and VTC from 0.62 to 0.73.

**Fig. 6:**
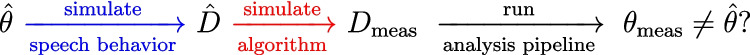
Process for assessing the impact of classification errors on a particular analysis pipeline. A true value $\hat{\theta }$ for a parameter of interest is drawn at random. The model of speech behavior is simulated given $\hat{\theta }$, which yields synthetic ground truth data, $\hat{D}$. The behavior of the algorithm is simulated, thus returning synthetic *algorithmic* data, $D\ne \hat{D}$. Finally, the analysis pipeline is run on $D_{\text {meas}}$. If $\theta _{\text {meas}}$ significantly departs from $\hat{\theta }$, then the process is sensitive to algorithmic bias

Most importantly, we compared manual vocalization count estimates (obtained by extrapolating partial human annotations) with the automated estimates derived before and after calibration. Prior to calibration, automated estimates are severely biased (Figs. 14 and 15, Appendix §A.2.3). Calibration increases the agreement between manual and automated vocalization count estimates, with statistically significant improvements for CHI, FEM, and MAL under both VTC and LENA™. For female adult speech, the ICC increased dramatically, from 0.15 to 0.85 in VTC and from 0.02 to 0.81 in LENA™. This is despite the fact that a perfect match cannot be expected, since manual estimates based on partial annotations do not provide ground truth values for whole recordings, and because of the stochasticity of algorithmic errors.

Finally, these validation strategies were complemented by a simulation study, which enabled us to assess the ability of the calibration procedure to reliably estimate vocalization counts and parameters of interest when the true values are known. To this end, we chose plausible values for every parameter of the model of speech behavior, then simulated 1000 observations (200 children $\times$ 5 recordings each). We simulated automated vocalization counts for each recording by modeling confusion errors (using a strategy described in detail in the next section) and applied the Bayesian calibration on these synthetic corpora. This revealed that Bayesian calibration systematically improves the accuracy of automated vocalization counts (Table 6, Appendix §A.2.4) and generally improves the estimates of quantities and effect sizes of interest (Fig. 17). The simulation study gives us an opportunity to assess whether the posterior distributions obtained via Bayesian calibration correctly reflect the uncertainty in the quantities of interest – specifically, in our example, the vocalization counts per synthetic recording – (Cook, Gelman, & Rubin, 2006). The posterior distributions are properly calibrated if their credible intervals achieve nominal coverage – that is, an X% credible interval contains the true value X% of the time. Calibration curves in Fig. 16 (Appendix §A.2.4) reveal that the posterior distributions are indeed very well calibrated, with perhaps the exception of male adult speech in VTC, where the posteriors are a bit too wide.

### An alternative to calibration: simulations for sensitivity analysis and null-hypothesis testing

Bayesian calibration can be computationally demanding, particularly for large datasets. Moreover, it is sometimes unnecessary: certain measurements are only marginally affected by classification errors, making the added complexity unwarranted. In such cases, simulation-based approaches are straightforward alternatives that can effectively assess the sensitivity of an analysis to classification errors or evaluate whether a finding could be explained by classification errors alone (see Fig. 6). Like Bayesian calibration, however, simulations still require (1) a model of speech behavior and (2) a model of the algorithm’s behavior.

In a simulation-based approach, the first step is to generate synthetic datasets reproducing key characteristics of the actual data to be analyzed (including the amount of participants and observations), by sampling from the assumed model of speech behavior. Synthetic data can be simulated by fixing the value of one or several parameters of interest, say $\hat{\theta }$ (for instance, by considering a null-hypothesis, $\hat{\theta }=0$). This gives $\hat{D}$, the synthetic “true” vocalization counts. The behavior of the algorithm itself is then simulated, by drawing samples from the corresponding model, yielding $D_{\text {meas}}$ (the vocalization counts as they would be reported by the algorithm). The resulting synthetic datasets can then finally be processed within an analysis pipeline (e.g., a linear regression), producing an estimate of $\theta _{\text {meas}}$. This estimate can be compared to the known true value ($\hat{\theta }$). This procedure can be repeated for different values of $\hat{\theta }$. If the difference between $\mathbb {E}(\theta _{\text {meas}})$ and $\hat{\theta }$ is generally negligible, then algorithmic errors do not seriously bias inferences. We illustrate this approach with the measurement of the proportion of female adult speech in Section “Anticipating biases with simulations”. Whenever simulations indicate significant vulnerability to classification bias, one can consider the Bayesian calibration procedure described above (Section §“Model of the algorithm’s behavior”) for deriving unbiased estimates of $\theta$. Alternatively, in null-hypothesis testing settings, simulations can assess whether a finding (e.g., a positive correlation between two speakers) is compatible with the effect of classification errors alone. We provide a Python package to facilitate the implementation of such simulations for VTC and LENA™, including tutorial examples in Python and R (Gautheron, 2025).

**Table 1:** Corpora used in the present analyses. Fausey-trio is only used for purposes of calibration (full recordings from this corpus are not considered in the model)

	Full recordings				Human annotations				
Corpus	# Child.	# Recs	Time (h)	Age-range (mo)	# Child.	# Recs	Time (h)	Age-range (mo)	Sampling strategy
bergelson	44	450	3600	6-17	10	13	5.0	7-17	Random
cougar	27	143	1144	2-67	26	27	6.5	25-37	High vol (CHI-adult)
kidd	96	554	4432	9-26	0	0	-	-	-
lucid	35	224	1792	11-32	10	10	5.0	11-31	Random
warlaumont	9	17	136	3-18	10	14	5.0	3-9	Random
winnipeg	6	13	104	2-19	9	10	5.0	2-32	Random
fausey-trio	0	0	-	-	23	23	1.1	6-12	High vol (MAL, OCH)

### Data

To inform the current exploration, we build on six corpora: Bergelson (English monolinguals, North America, Bergelson, 2015), Cougar (English monolinguals, North America, VanDam, 2018a), LucCiD (English monolinguals, United Kingdom, Rowland, Bidgood, Durrant, Peter, & Pine, 2018), Kidd (English, predominantly monolinguals, Australia, Donnelly & Kidd, 2021), Warlaumont (English-Spanish bilinguals, North America, Warlaumont, Pretzer, Mendoza, & Walle, 2016), Winnipeg (mostly English monolinguals, with some French spoken, North America, McDivitt, & Soderstrom, 2016), Fausey (English monolinguals, North America, Mendoza, & Fausey, 2022; Fausey, 2018). A conjunction of imperatives constrained dataset selection: the data had to be longitudinal and to simultaneously contain the raw recordings, together with LENA *and* VTC annotations. Additionally, we only included audio data between 10:00 am and 6:00 pm, and we excluded recordings which do not cover this time range entirely. This allows for more consistent comparisons across data points. The selected audio amounts to $\sim 11\,200$ hours total.

The two algorithms we consider differ on many aspects, from their training data to their architecture, but for the purposes of conciseness, we focus here on the aspect of their behavior most likely relevant to the question at hand: Their propensity to miss vocalizations, and to confuse or co-activate speaker classes. LENA™ uses a Dirichlet Process Gaussian Mixture Model to classify audio frames into categories including key child speech, other speakers, noise, and silence. The system prioritizes precision over recall, meaning it tends to be conservative in identifying vocalizations to avoid false positives. VTC is an open-source neural network-based alternative to LENA that detects the same four speaker types but can handle overlapping speech and has been trained on multilingual data. Unlike LENA, VTC balances precision and recall by maximizing F-score, resulting in higher recall but lower precision. For detailed technical specifications and implementation details, see Xu et al. (2008), Gilkerson et al. (2017) for LENA and Lavechin et al. (2020), Lavechin et al. (2025) for VTC (Table 1).

Human annotation was available for snippets from five of our six key corpora, covering only 0.23% of the total audio duration. Most of our human annotation data (totaling 20h) comes from the ACLEW collaboration, and has been documented in Soderstrom et al. (2021). Annotators were trained until they met stringent criteria on a gold standard, and all labs used the exact same annotation manual (the ACLEW DAS template, Casillas et al., 2017), including definitions of what constitutes a vocalization. We employed here the so-called “random sample”: Fifteen two-minute clips were randomly sampled from one recording from each of 9–10 children in four English-spoken corpora. The use of random sampling safeguards against any bias in data selection coming from the use of an algorithm. An additional 6.5 h comes from the VanDam-5-min corpus (Vandam, 2018b; Carns, 2015), which has been less overtly documented, but the description on Homebank (VanDam, 2018a) provides sufficient details by explaining that three non-consecutive five-minute sections were sampled that had the highest child–adult conversational turns according to the automated LENA™ analysis. Annotators had access to LENA™ segmentation and could correct it, but did not have to, as their priority was to produce orthographic transcriptions of what was said. To further inform our analyses, we also included in-house human annotations on Fausey-Trio (Fausey, 2018), a seventh corpus that is otherwise not included in our analyses. Human annotation of this corpus was done independently of the present paper, with the purpose of contributing to a dataset with greater representation of male adults and other children. To this end, audio was sampled using 15-s-long snippets and a loudness-based filter (i.e., independent from both LENA™ and VTC). Human annotators first listened to the snippets and only annotated them if there was at least one vocalization by adult males and/or other children (i.e., snippets with only key child and/or female adult vocalizations were not annotated). The annotation followed a simplification of ACLEW DAS focused only on segmentation, without transcription. In sum, most of the human annotation was done independently of the algorithms whose behavior is studied in this paper; and by design all four speaker classes are present in the human-annotated data. In total, the 27.6 h of audio annotated by a human, VTC, and LENA™ yielded 6638 $\times$ 15-s clips to be used for calibration purposes.^11^

## Results

We now turn to our main goal, the study of the effect of classification errors on downstream statistical analyses. In Section §“Comparing LENA™ and VTC against manual coding”, we start by comparing the two speech-detection algorithms in our case-study (VTC and LENA™) against manual annotations. We will see that the two algorithms make errors that can explain the discrepancies between correlation estimates derived from human, VTC, and LENA™ annotations (first evoked in Fig. 3, Section §“The case of voice type classifiers when describing early language acquisition”). In Section §“Effect of classification bias on downstream analyses and measurements”, we apply the Bayesian calibration strategy to our dataset. We compare the estimates of a variety of quantities and effect sizes relevant to language acquisition, using either manual annotations, or annotations from each algorithm, before and after applying our calibration strategy. We will see that without calibration, VTC and LENA™ produce generally inconsistent estimates; using our calibration procedure, the tension is often reduced; in addition, calibrated estimates can significantly deviate from the naive estimates. However, in some cases, Bayesian calibration failed to resolve the disagreement between VTC and LENA™. Finally, in Section §“Anticipating biases with simulations”, we show how simulations can help anticipate and diagnose biases resulting from algorithmic errors.

### Comparing LENA™ and VTC against manual coding

This section uses our joint model to assess how LENA™ and VTC align with manual coding. This analysis addresses two questions: (1) how accurately do the automated methods detect speakers’ vocalizations compared to manual coding, and how consistently they behave, and (2) how do the models’ classification errors explain the distortions of correlations between speakers shown in Fig. 3.

**Fig. 7:**
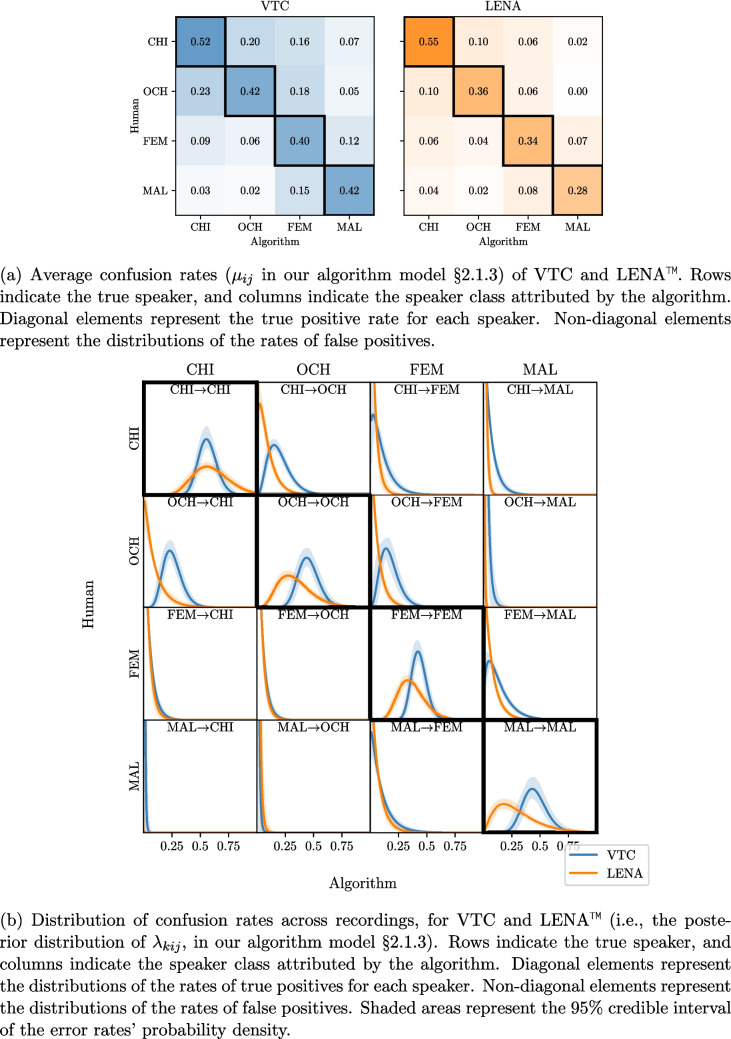
Confusion rates of VTC and LENA™

By fitting the calibration model introduced in Section §“Model of the algorithm’s behavior” to the calibration data (i.e., clips of audio for which human annotations are available), we can evaluate the accuracy of classifications against human annotation. These can be represented in the form of confusion matrices, as shown in Fig. 7a. The confusion matrices show that both algorithms confuse certain pairs of speakers more often: CHI and OCH (i.e., children) on the one hand, and FEM/MAL (i.e., adults) on the other. In addition, female adults are more often confused with children (and vice versa) compared to male adults. Comparing the two algorithms, LENA™ achieves fewer false positives compared to VTC, but also exhibits lower true positive rates, in line with previous observations that LENA™ has a generally higher precision but lower recall (Lavechin et al., 2025).

Average confusion rates, however, are not sufficient metrics for comparing the merits of multiple classifiers. Another criterion is whether the performance of these algorithms is *consistent* throughout recording conditions. Imagine a classifier with a very consistent detection rate of 50%. The actual number of events could still be estimated precisely by multiplying the number of detected events by 2. Now imagine a different classifier that has lower confusion rates but more variable performance, for instance, with a detection rate varying widely between 50 and 70%; although it is more accurate on average than the first classifier (because the detection rate is higher), its calibration would be much trickier because one cannot simply multiply detected events by two. To compare the consistency of LENA™ and VTC, Fig. 7b shows the distribution of confusion rates throughout recordings. The distributions of the true-positive rates (on the diagonal) are more peaked for VTC than for LENA™, indicating that the former’s recall rates are more consistent across recordings. The shading behind each of the curves in Fig. 7b also gives us another important piece of information, as it represents the uncertainty about the underlying distribution of confusion rates. We note that this uncertainty is higher for underrepresented speakers (other children, and especially male adults).

Having established how LENA™ and VTC differ in their classification accuracy against manual coding, we now turn to question (2): how do these classification errors explain the correlation distortions observed in Fig. 3, Section §“The case of voice type classifiers when describing early language acquisition”? Turning back to this figure, we indeed observe that VTC and LENA™ report higher correlations between CHI, OCH and FEM, MAL than humans. We also find that VTC and LENA™ report higher correlations between CHI/OCH and FEM than between CHI/OCH and MAL, again in line with what we expect from classification errors. In addition, the confusion matrix shows that VTC exhibits higher rates of false positives than LENA™ generally. VTC reports higher correlations between speakers than LENA™ across the board, as expected from diarization errors; this is true even when correlations are computed at the levels of recordings and children (Figs. 24 and 25, Appendix §A.6).

These findings are consistent with the distortions of correlations observed in the previous section. First of all, VTC shows higher correlations across the board, consistent with its higher false-positive rates. In addition, regardless of the algorithm, the distortion of correlations is generally greater for pairs of speakers that are often confused with one another (e.g., CHI/OCH, OCH/FEM, or FEM/MAL). This is true for LENA™ as well, despite its lower rate of false positives. While this establishes the biasing effect of speaker confusion on correlations between speakers, the next section shows that this issue also affects measurements and inferences, implying quantities more obviously relevant to language acquisition.

### Effect of classification bias on downstream analyses and measurements

**Fig. 8:**
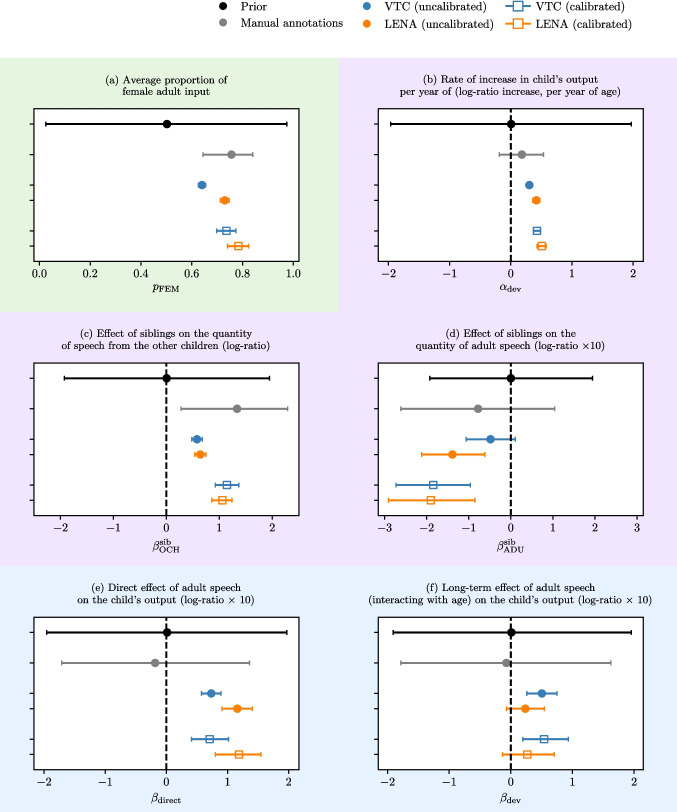
Comparison of effects’ sizes derived with manual annotations alone (in gray) and automated annotations (in colors), without any calibration and with calibration. The prior distribution ($\mathcal {N}(0,1)$ or $\mathcal {U}(0,1)$, depending on the variable support) is shown in black for purposes of comparison. We distinguish three types of measurements: direct measurements of speech quantities (**a**); measurements of the effect of an independent variable on speech quantity (**b**, **c**, **d**); and measurements of the effect of a quantity of speech on another quantity of speech (**e**, **f**). Numerical values are reported in Appendix §A.2.5, Table 7

In what follows, we report the effect of classification bias on six measurements of variables and effects relevant to language acquisition, using the model detailed in Section §“Model of speech behavior” (see Fig. 8). These are: (a) the proportion of female adult input; (b) the effect of age on children’s speech output; (c) the effect of siblings on the quantity of input from other children and (d) from adults; (e) the direct effect of adult speech on children’s output; (f) the long-term effect of adult input on children’s output. Each measure is estimated using manual annotations alone, automated annotations without any calibration, and automated annotations with Bayesian calibration. Results are grouped by type of measurement.

In every case, the prior distribution (in black) reveals the initial hypothesis with respect to each measurement (the Bayesian prior), before considering any kind of data. The next set of observations (in gray) pertains to manual annotations. Since manual annotations are available only for portions of a limited subset of recordings, they result in wide credible intervals largely reflecting the prior, indicating that little to nothing could be learned. Automated annotations are associated with much narrower credible intervals in all cases, demonstrating the benefits of machine learning classifiers in harnessing more data from which to estimate an effect. Finally, we show posteriors extracted from the joint model, which separates the contributions to vocalization quantities from the model of speech behavior from those due to confusion, captured by the model of algorithm behavior. The calibrated estimates are associated with larger credible intervals, reflecting our uncertainty in light of the stochastic nature of the algorithms’ behavior.

#### What is the contribution of female adults to children’s speech input?

First, we consider the proportion of input attributed to female adult voices (Fig. 8a). The prevalence of female versus male adult speech is relevant to a range of disciplines, feeding theoretical discussions on variation in parental investment as a function of family organization (e.g., Cassar, Cristia, Grosjean, & Walker, 2025) as well as interventions geared at greater involvement of fathers (e.g., Ferjan Ramírez, Hippe, Correa, Andert, & Baralt, 2022). However, confusion between different speakers may distort our estimates of female input proportion (Fig. 2a). For instance, vocalizations from children may be falsely attributed to adults; and vocalizations from male and female adults could be mistaken with each other, possibly at different rates. In Fig. 8a, we find that LENA™ and VTC yield incompatible measurements of the proportion of female adult speech, with VTC probably underestimating it. After calibration, VTC and LENA™ estimates are closer to each other.

#### Do children vocalize more with age?

Next, we consider the rate of increase in children’s output with age (Fig. 8b). Age-related increases in typically developing children’s vocal production in long-form recordings have been widely documented, using both LENA™ (e.g. Bergelson et al., 2023) and VTC (e.g., Hervé, François, & Prevot, 2024). These changes could potentially be due to various reasons, including simple maturation – by which we mean processes that occur independently from exposure or learning. For instance, the emergence of canonical syllables in children’s production is independent of auditory experience because deaf and hearing infants start babbling at roughly the same ages; but canonical syllables are more frequent and diverse in hearing than deaf children, suggesting that a process in addition to simple maturation is necessary for the latter changes (Oller, Eilers, Steffens, Lynch, & Urbano, 1994). Age-related increases in vocal production are thought to reflect not simply maturational changes, but actually improvements in children’s language skills, based on correlations between vocalization quantity and standardized measures of language development (a meta-analysis in Wang et al., 2020) and the observation that children with atypical development show different age-related changes (Warlaumont, Richards, Gilkerson, & Oller, 2014a; Hamrick, Seidl, & Kelleher, 2023). Figure 8b shows that manual annotations are too sparse to measure the increase in children’s output with age confidently. By contrast, automated annotations from LENA™ and VTC produce highly confident but non-overlapping estimates. Calibration increases the developmental effect of age and reduces the gap between the two algorithms. Most likely, when classification errors are unaccounted for, speech output is contaminated with input speech that is less sensitive to the child’s age, thus damping out the estimated effect of age.

#### How do siblings affect children’s language input?

Third, we consider the effect of siblings on the quantity of input children receive from other children (Fig. 8c) and adults (Fig. 8d). Among certain populations, speech from other children in the main exposure to language in infants (Cristia et al., 2023), and the contribution of siblings (as opposed to e.g. non-kins) may vary in rural and non-rural contexts. In our data, automated annotations without calibration show that children with no siblings receive about $\sim 40\%$ fewer vocalizations from other children than children with siblings. This difference is implausibly low in the corpora under consideration in which siblings are expected to be, by far, the primary source of exposure to speech from other children. By contrast, the calibrated estimate ($\sim 67\%$ less input from children for participants without siblings) is much more plausible. Without proper calibration, both algorithms under-estimate the effect size by a factor of two. Thus, without calibration, the contribution of non-kin children cannot be reliably evaluated, which is a problem in populations where it is substantial. We then considered the effect of siblings on the input received from adults. Using 1-h-long home-recorded videos, Laing and Bergelson (2024) find that children with more than one sibling receive less input from their caregivers. This result is consistent with “the resource dilution” model, a hypothesis that was put forward to explain a pattern of lower educational attainment as a function of sibship size by arguing that, as the number of children increases in a household, the main holders of intellectual resources (the adults) have to split them among the children, resulting in fewer resources per child (e.g., Kalmijn & van de Werfhorst, 2016). We tried to replicate this finding using our comprehensive automatic annotations of child-centered long-form recordings. First of all, the effect of siblings on adult input is imperceptible with manual annotations alone. Automated annotations provide strikingly different estimates; while LENA™ finds that siblings reduce the amount of input afforded by adults to the child, VTC estimate is compatible with the null hypothesis (95% credible level). After calibration, the estimate of this effect is much larger, pointing to a 20% reduction in adult input among children with siblings, and consistent across VTC and LENA™.

#### Does hearing more speech make children talk more, in that recording or in the long run?

The final examples consider the measurement of the correlation between “input” (speech heard by children) and “output” (how much speech they produce) (Fig. 8e, f). These have been previously approached in many different ways (as discussed in the meta-analysis by Anderson, Graham, Prime, Jenkins, & Madigan, 2021), including using daylong recordings (as in the meta-analysis by Coffey and Snedeker, 2024). For example, Bergelson et al. (2023) in the most diverse LENA™ study to date, reported a strong association between the quantity of adult speech afforded to children and the rate at which their speech production increases over time, a finding that is worth attempting to replicate. In that study, both measures were derived from the same audio recordings and analyzed with LENA™ software, with CVC predicted by adult vocalization counts (AVC). Of course, an association between input and output cannot be easily interpreted as indicating a causal relationship. For instance, Bergelson et al. (2023) raise concerns a positive correlation may (partially) reflect shared genetics between the adults and children recorded^12^. As shown in Fig. 2b, speaker classification errors could constitute an additional cause of confound, which, to our knowledge, has not been discussed. In our case, we distinguish the recording-level effect of input on output (surfacing as higher amounts of child speech in recordings with more input from adults, *ceteris paribus*), from the long-term developmental effect of input on child’s speech, resulting from sustained exposure to higher input over time. No effects are found when using human annotations alone. Without calibration, VTC and LENA™ find positive effects, although their estimates are non-overlapping. After calibration, the effects remain roughly unaltered (except for larger uncertainties), and VTC/LENA™ continue to disagree. Persisting discrepancies between LENA™ and VTC indicate that the algorithms differ in ways unaccounted for by the calibration model.

In our typology of measurement, calibration works best for effects of a known variable on a speech quantity: intuitively, independent variables can help the model discriminate between spurious and actual correlations. Calibration was less useful for direct correlations between quantities of speech. One potential reason is that such correlations are most directly affected by classification errors and thus harder to tell apart, especially if the size of the actual effect is comparable in magnitude to the confusion rates.

**Fig. 9:**
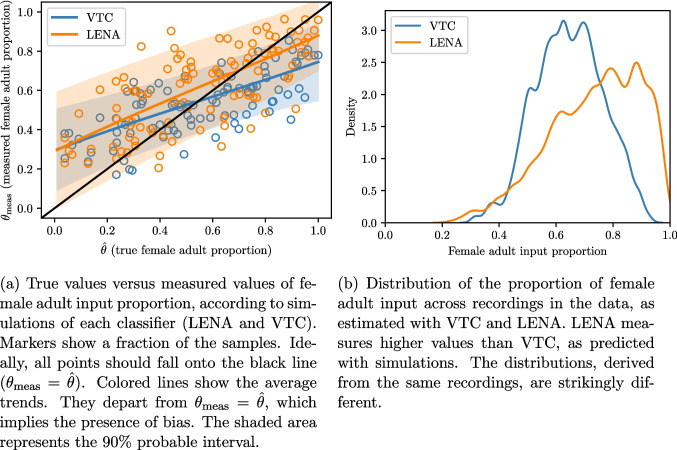
Impact of speaker confusion on measurements of female and male adult speech

### Anticipating biases with simulations

Classification bias can distort statistical measurements to varying extents. How to predict the level of sensitivity of a particular measurement to classification errors? This question can be answered with the simulation strategy proposed in Section §“An alternative to calibration: simulations for sensitivity analysis and null-hypothesis testing”. For instance, let us consider the relative contribution of female adults to adult input. We generate random datapoints representing the true amount of vocalizations from each speaker (CHI, OCH, FEM, and MAL) using the following generative process:

$$\begin{aligned} \text {CHI}&= 1500\\ \text {OCH}&= {\left\{ \begin{array}{ll} 0 & \text {with probability } 1/2\\ 1000 & \text {with probability } 1/2 \end{array}\right. }\\ \text {FEM}&= 3000 \times p\\ \text {MAL}&= 3000 \times (1-p)\\ p&\sim \text {uniform}(0,1) \end{aligned}$$

The total quantity of adult input (FEM+MAL) is fixed. The proportion of female adult input is represented by a parameter $\hat{\theta }=p$ drawn uniformly between 0 and 1. We can then simulate the algorithm’s output for each generated sample, and compare the measured fraction of female adult speech $\theta _{\text {meas}}$ to the true value $\hat{\theta }=p$. The results are shown in Fig. 9a (using 2000 samples). They demonstrate that estimates of the proportion of female adult speech are biased, especially for extreme proportions (close to 0 or 1). Moreover, LENA™ estimates higher fractions of female adult speech than VTC, and tends to overestimate female adult speech up to $p\sim 0.75$. This is probably because it was calibrated on training data dominated by female adult speech, and because the algorithm relies heavily on speakers’ average prevalence to inform its classification boundary. Simulations also reveal the variance in measurements (as indicated by the shaded areas in Fig. 9a). LENA™ has generally higher dispersion. The reliability of this simulation approach can be assessed by comparing these predictions to actual data. Figure 9b shows the distribution of the proportion of female adult input estimated by VTC and LENA™ across the same corpus of audio recordings. The distribution obtained with LENA™ is shifted to the right (i.e., LENA™ reports higher proportions) and it has larger variance than the distribution given by VTC, as predicted by the simulations. Moreover, VTC saturates around $p=0.9$, again in accordance with the simulations.

## Discussion

### Empirical findings

We investigated the statistical biases resulting from errors in automated classification. Although we validated our approach on long-form recordings in the context of language acquisition research, we note that it is conceptually applicable to any classification algorithm that detects events (in speech or other domains). We found evidence of spurious associations between speakers resulting from “biasing paths” opened up by speaker misidentification. Differences in correlation estimates across LENA™ and VTC appear consistent with expectations given their confusion rates (e.g., VTC has higher confusion rates and reports higher correlations between speakers). Using simulations, we found even more evidence that classification errors can account for certain discrepancies between LENA™ and VTC. For instance, simulations informed by calibration data correctly predict that VTC underestimates the proportion of speech from female adults, more than LENA™, and produces less dispersed estimates across recordings.

We also studied how our Bayesian calibration approach could be used to alleviate biases. We found that our approach produces wider credible intervals, better reflecting our true uncertainty about the final measurements. Additionally, this approach reduces disagreement in inferences across the two algorithms for almost all research questions we examined. For instance, after calibration, LENA™ and VTC find a consistent negative effect of siblings on adult input, 20–80% stronger than measured prior to calibration. However, in the case of recording-level association between adult input and child output, Bayesian calibration failed to reduce the disagreement between LENA™ and VTC. In this particular case, our approach fails to resolve the gap between VTC and LENA™. This suggests that there remain differences in these algorithms that our model of algorithm behavior does not account for, and invites further work to complexify it. This exception notwithstanding, we provide ample evidence that our proposed approach can improve inferences.

Below, we unpack the implications of our work (§“Implications and recommendations”), for statistical inference with machine learning classifiers in behavioral science (§“Implications for statistical inference with machine learning classifiers”) and for child-development/language acquisition research in particular (§“Implications for child development and language acquisition research”). Finally, we review present limitations of Bayesian calibration and opportunities for future works (§“Limitations of Bayesian calibration and opportunities for future works”).

### Implications and recommendations

#### Implications for statistical inference with machine learning classifiers

Although our main goal in this paper is to lay out some ways in which classifier errors may affect scientific conclusions rather than provide a foolproof, out-of-the-box solution, we feel that it would be inappropriate to end the paper without attempting to produce a set of actionable recommendations.

First of all, standard measures in psychology such as accuracy, reliability, and validity are insufficient for addressing biases resulting from classification errors: two measures (e.g.: voc counts CHI, voc counts FEM) can be individually reasonably accurate (i.e., each measure is strongly correlated with human judgment), reliable (i.e., each measure is stable across repeated measurements), and valid (i.e., each measure is strongly correlated with independent relevant metrics), and nevertheless spuriously correlate to each other. Instead, we recommend using simulations to assess the impact of algorithmic errors on a measurement of interest, as we have illustrated with the proportion of female adult speech. Simulations are relatively inexpensive to run and easy to implement. They may be used in null-hypothesis testing contexts, for assessing whether a finding is compatible with the absence of an effect, once classification errors have been taken into account. For the specific case of LENA™ and VTC, we facilitate the adoption of this approach by introducing a Python package simulating their outputs given synthetic ground-truth data (Gautheron, 2025). In addition, experienced users may consider Bayesian calibration, which can directly recover unbiased estimates. However, this strategy remains experimental and comes at the expense of higher technical and computational costs.

As a robustness check, one may execute an analysis pipeline using annotations from different classifiers. This can provide an indication of the analysis’s sensitivity to classification errors. For instance, LENA™ and VTC often yield non-overlapping estimates, given the differences in how they balance recall and precision (see Fig. 7a). This strategy, however, is not nearly as reliable as the appeal to simulations, because multiple classifiers can misbehave in similar ways, achieving mutually compatible but nevertheless biased estimates (see, e.g., Fig. 8c).

We would like to conclude with a recommendation to producers of machine learning algorithms. As we have seen, LENA™ and VTC behave differently in part due to a different approach towards the precision/recall trade-off. The optimal balance of recall and precision might depend on the task at hand, and the relative harm of false positive versus false negatives (Silva Filho et al., 2023). We therefore suggest that computer scientists allow the users of their algorithms to re-adjust the recall/precision trade-off according to their need, based on the confidence scores of the predictions^13^.

#### Implications for child development and language acquisition research

##### Re-assessing LENA™ and VTC

Besides the observation that statistical measures of interest for child development are distorted by classification errors, this work offers a new opportunity to assess the relative merits of LENA™ and VTC. On average, because of a stronger emphasis on precision over recall, LENA™ may seem less subject to classification bias. However, we observed that LENA™’s behavior is more variable, which increases measurement noise and renders calibration more challenging; and its inability to support overlapping speech is harder to model and account for rigorously. That said, LENA™’s algorithm has been stable for over ten years, in principle allowing greater comparability across studies. In contrast, VTC is an open-source algorithm, which benefits from improvements incorporating state-of-the-art solutions in speech processing (Kunze et al., 2025). This is both a strength and a limitation. Recent developments (VTC 2.0, Charlot et al., 2025) radically improved precision and recall, drawing close to human-human agreement for the MAL and FEM categories and closing the human-algorithm performance gap for OCH. While this improves accuracy, it means users will need to revisit the current calibration work, as our above report may not generalize to the new VTC. We hasten to indicate that two of the authors of the present paper are involved in both the original VTC and ongoing work, which may constitute a conflict of interest biasing our perception. We thus strongly encourage readers to peruse our results carefully to make up their own minds.

##### Data collection

Considering the amount of manual annotations required to implement the simulation or the Bayesian calibration approach, researchers may find it necessary to momentarily rely on our own estimates of the confusion matrices of LENA™ and VTC (as provided by our package Gautheron, 2025), even though these confusion rates were derived from non-representative corpora (urban, English-speaking populations). Nevertheless, several reasons may warrant further manual annotation efforts.

First of all, there are a number of outstanding questions that will require either coordinated annotation efforts (as in Casillas et al., 2017) or sharing facilitated by standardized solutions (Gautheron, Rochat, & Cristia, 2022). We observe a great deal of variation in confusion rates, but at present lack sufficient data to find the sources of this variation. Indeed, preliminary experimentation suggested our models could not converge with less human annotation than the 28 h we included. We believe it would be desirable to multiply the quantity of human annotation by a factor of 4, so as to be able to subset it at least into 25%, 50%, and 75%, and thus assess how data quantity affects uncertainty. Since it took four teams 3 years to annotate the 20h in Bergelson, Lucid, Warlaumont, and Winnipeg (Soderstrom et al., 2021), it may take several years to quadruple that data amount.

When possible, we think it may be strategic to sample data in ways that inform both sections of the joint model. Regarding the speech behavior section of the joint model, and as explained above, other researchers may propose alternative (simpler or more complex) models of speech behavior, which would have effects on the data required. Nonetheless, assuming our model of speech behavior, the largest gap is the absence of human annotation of samples extracted from multiple recordings within each child; that is, the ACLEW project drew multiple samples from a given child, but all of them came from the same recording day, which misses both random cross-recording variation and potentially systematic longitudinal variation.

Regarding strategic sampling to inform the algorithm behavior section of the joint model, most of the human annotation data we used was based on random sampling, because we wanted to avoid potential biases emerging from sampling mainly from sections where conversation was more prevalent. However, future iterations could benefit from a better understanding of how different auditory contexts affect both the algorithms and the human annotators. There is already some work looking at the agreement across humans and algorithms as a function of diverse factors ranging from background noise to the key child’s age. For instance, Peurey et al. (2025) discusses several factors that could explain variation in performance in voice type classifier algorithms, while also providing evidence that only some of them act as predicted. We extended these analyses in Appendix A.3, which shows little compelling evidence that confusion rates vary as a function of child age or corpora. Nonetheless, this work still does not distinguish between increased errors in the algorithm versus increased errors in human annotation. To this end, annotating data to increase overlap between annotators would be necessary, as would extensions to the algorithm behavior model to capture this variance.

### Limitations of Bayesian calibration and opportunities for future works

Bayesian calibration leverages both human and automated annotations to produce unbiased estimates with credible intervals reflecting our true uncertainty given the stochasticity of the underlying machine learning algorithms. Consistent with prior work, we indeed find that more robust results are obtained through the *combination* of human and automated annotations (Angelopoulos et al., 2023b; Teblunthuis et al., 2024). In contrast to prediction-powered inference (Angelopoulos et al., 2023b), Bayesian calibration is effective even with very few human annotations (equivalent to 0.2% of the audio in our case). It is also much more flexible, since it decouples the model of the algorithm from the assumed behavioral model, allowing both to be refined in parallel. However, in contrast to Angelopoulos et al. (2023b), Bayesian calibration requires assumptions about the behavior of the algorithm itself. Since these assumptions can be too simplistic or incorrect, this provides less guarantee in general.

Interestingly, Bayesian calibration has the potential to integrate predictions from different classifiers into a single analysis, whether these algorithms cover disjoint or overlapping portions of the data. While this requires minimal work in principle (see Appendix §A.7 for an example), we found that our approach was too slow to integrate both VTC and LENA™ annotations in our large corpus. However, we believe further optimization is possible. Alternatively, approximate inference methods such as Variational Inference may provide further speedups, at the expense of accuracy.

Additionally, Bayesian calibration may achieve efficiency gains by incorporating additional predictors of the confusion rates. For example, confidence scores^14^, when provided by machine learning classifiers, could also be used as covariates in our calibration model. Similarly, automatically inferred estimates of audio quality (e.g., signal-to-noise ratios or reverberation levels) are also correlated with accuracy and could act as complementary covariates (Kunze et al., 2025; Lavechin et al., 2023).

Finally, some researchers study properties of conversations (Abney, Warlaumont, Oller, Wallot, & Kello, 2016), such as the probability of adults reinforcing children’s speech-like vocalizations (Warlaumont, Richards, Gilkerson, & Oller, 2014b), or the timing of inter-speaker turns (Ritwika et al., 2025). Extending our models to address the temporal nature of speech will be challenging. In our Bayesian framework, this would require estimating the probability that a true sequence of vocalizations (e.g., adult, child, adult, child, ...) is detected as any other sequence (e.g., adult, adult, child, ...). The space of possible sequences is combinatorially large, limiting this approach to short sequences. Time-coding precision, which we neglected, may also become critical. Finally, algorithms distort sequences in complex ways—through constraints; LENA™ imposes a minimum duration on vocalizations, and is unable to handle overlaps, while VTC overproduces vocalizations with durations that are multiples of 250 ms. Modeling such distortions is challenging but important for certain applications.

## Conclusion

With this paper, we aimed to bring attention to the potential downstream consequences of classification and segmentation errors made by machine learning algorithms. As research attempts to capture behavior through denser and more ecological datasets, machine learning will become increasingly prominent. We thus do not recommend abandoning it, but rather increasing our understanding of where and how this tool’s imperfection can affect our scientific conclusions, even if our data modeling needs to become more complex in order to account for those imperfections. The proposed Bayesian calibration approach is a first step in this direction.

## Supplementary Information

Below is the link to the electronic supplementary material.

Supplementary file 1 (pdf 14396 KB)

## Data Availability

The datasets analyzed during the current study are not publicly available. However, the code includes synthetic data and routines to generate synthetic datasets, which allows readers to check the correctness of their implementation. In addition, the code can be run on any dataset formatted according to the ChildProject guidelines (Gautheron et al., 2022).

## Footnotes

1 LENA™ also returns other classes, such as TV/electronic noise. Since these have been less often the target of methodological work and are less commonly used in scientific research by and large, we do not consider them here.

2 As an example, the data we introduce in the Methods section contains a day-long recording where LENA™ finds fewer than 200 vocalizations from male adult(s) and VTC finds more than 2000

3 Their approach relies on a *rectifier*, i.e., a function that compensates for measurement errors in the estimator of a quantity of interest (e.g., a linear or logistic regression coefficient) by leveraging manual annotations. However, deriving the rectifier is not a trivial task for an arbitrarily complex model. For very simple models, ready-to-use implementations have become available (Angelopoulos, Bates, Fannjiang, Jordan, & Zrnic, 2023a; Salerno et al., 2025). Efforts to enhance the flexibility of this line of approach are ongoing (Miao & Lu, 2024).

4 The authors present their method as an improvement over previous approaches such as Multiple Imputation (MI). In MI, multiple analyses are performed and pooled together given different imputations of missing or noisy data (Blackwell, Honaker, & King, 2017).

5 That is, the posterior density of the true quantity of vocalization can never be constructed without assuming a model of speech behavior (because $P(\hat{D}|D_{\text {meas}})\propto P(D_{\text {meas}}|\hat{D})\times P(\hat{D}|\theta )P(\theta )$, where the first factor is informed by the algorithm behavior model and the second is informed by the speech behavioral model).

6 We assume $\tau \sim \text {Exponential}(1)$, such that the model can accommodate both under- and over-dispersion. We use the Stan implement of the double Poisson distribution proposed in Pustejovsky (2024). We find $\tau \sim 1.4$ for VTC and $\tau \sim 1.8$ for LENA™, corresponding to underdispersion.

7 $\alpha$ is difficult to identify, especially for low values of $\mu$. Ideally, we would need more extensive human annotations within each manually annotated recording. In this work, we limited ourselves to extant annotations.

8 See https://mc-stan.org/docs/stan-users-guide/latent-discrete.html.

9 The F-score is the harmonic mean of precision ($\text {true positives}\over \text {true positives+false positives}$) and recall ($\text {true positives}\over \text {true positives+false negatives}$).

10 Instead, as a mere starting point, we suggest a simple, albeit naive step for accepting redundant annotations by making the following change to the model: when a unique audio clip *k* has been annotated by $A>1$ individuals, assume that there exists $1\le a\le A$ such that $\boldsymbol{v}_{\boldsymbol{ka}}$ are the true vocalization counts for this clip, and draw algorithmic counts accordingly. Marginalizing over all possible *a* gives:

5 $$\begin{aligned} p(\boldsymbol{n}_k) = \sum _{a=1}^A p(\boldsymbol{n}_k|\boldsymbol{v}_{ka})p(\boldsymbol{v}_{ka}) \end{aligned}$$

This minimal form of aggregation, however, does not dispense with the assumption that at least one annotator is correct.

11 While assessing the impact of the recording environment on confusion rates (Appendix §A.3, Fig. 22), we consider annotations from corpora outside this table. To avoid reader confusion, those corpora and annotations are introduced in the relevant Appendix.

12 This explanation would only be partial, since input is required to acquire language, and that input can have effects independent of shared genetics between mother and child (Coffey, Shafto, Geren, & Snedeker, 2022).

13 For instance, VTC computes an evidence score for each class (each speaker) at each audio frame, which is a number between 0 and 1. In a second step, VTC decides if a class is active or not depending on whether the evidence score exceeds a certain threshold (which, in the case of VTC, is chosen to maximize the F-score, but could also be optimized differently)

14 Whether or not these have been calibrated themselves, in the sense of Guo, Pleiss, Sun, and Weinberger (2017).

## Code availability

The code of our models and analyses is available at: https://gin.g-node.org/LAAC-LSCP/speaker-confusion-model. The Python simulation package is available at https://github.com/LAAC-LSCP/diarization-simulation.
