# Supplementary material for: Classification errors distort findings in automated speech processing: Examples and solutions from child-development research
Source: Behav Res Methods. 2026 May 18;58(6):168. doi: 10.3758/s13428-026-03029-6 (PMC13183756; doi:10.3758/s13428-026-03029-6)
Supplement: Supplementary file 1 — (pdf 14396 KB) [file 13428_2026_3029_MOESM1_ESM.pdf]

A Supplementary materials

A.1 Models of speech behavior

A.1.1 Main model

For each recording k of child c, the vocalization counts are modeled as:

$$v_{k,\text{CHI}}^{\text{recs}} \sim \text{Gamma}\left(\alpha_{\text{child}}^{\text{CHI}}, \frac{\alpha_{\text{child}}^{\text{CHI}}}{\mu_{k,\text{CHI}}^{\text{rec}}}\right) \tag{6}$$

$$v_{k,s}^{\text{recs}} \sim \text{Gamma}\left(\alpha_{\text{child}}^{s}, \frac{\alpha_{\text{child}}^{s}}{\mu_{c,s}^{\text{child}}}\right), \quad s \in \{\text{FEM}, \text{MAL}, \text{OCH}\} \tag{7}$$

The child's expected vocalization rate incorporates age effects and the influence of adult speech:

$$\mu_{k,\text{CHI}}^{\text{rec}} = \mu_{\text{CHI}}^{\text{pop}} \exp\left(\alpha_c^{\text{dev}} \cdot \frac{\text{age}_k}{12} + \beta^{\text{dev}} \cdot \frac{\text{age}_k}{12} \cdot \frac{\mu_{c,\text{ADU}}^{\text{child}} - \mu_{\text{ADU}}}{\sigma_{\text{ADU}}} + \beta^{\text{direct}} \cdot \frac{v_{k,\text{ADU}}^{\text{recs}} - \mu_{c,\text{ADU}}^{\text{child}}}{\sigma_{c,\text{ADU}}^{\text{child}}}\right) \tag{8}$$

At the child level, for children with known sibling status:

$$\mu_{c,\text{OCH}}^{\text{child}} \sim \text{Gamma}\left(\alpha_{\text{pop},d}^{\text{OCH}}, \frac{\alpha_{\text{pop},S_c}^{\text{OCH}}}{\mu_{\text{OCH}}^{\text{pop}} \exp(S_c \beta^{\text{OCH}})}\right) \tag{9}$$

$$\mu_{c,s}^{\text{child}} \sim \text{Gamma}\left(\alpha_{\text{pop},d}^{s}, \frac{\alpha_{\text{pop},S_c}^{s}}{\mu_{s}^{\text{pop}} \exp(S_c \beta^{\text{ADU}}/10)}\right), \quad s \in \{\text{FEM}, \text{MAL}\} \tag{10}$$

where $S_c = 1$ if child has siblings, $S_c = 0$ otherwise.

For children with unknown sibling status, the model uses a mixture, marginalizing over the cases $S_c \in \{0, 1\}$, with probability p^{sibs} and $1 - p^{\text{sibs}}$ respectively.

The population-level parameters have the following priors:

$$\mu_s^{\text{pop}} \sim \text{Gamma}(2, 8) \quad (\text{prior mean} = 250 \text{ vocs/hour}) \tag{11}$$

$$\alpha_{\text{pop},d}^{s} \sim \text{Gamma}(8, 1) \tag{12}$$

$$\alpha_{\text{child}}^{s} \sim \text{Gamma}(4, 1) \tag{13}$$

Developmental effects are modeled with:

$$\alpha_c^{\text{dev}} \sim \text{Normal}(\alpha^{\text{dev}}, \sigma^{\text{dev}}) \tag{14}$$
$$\alpha^{\text{dev}} \sim \text{Normal}(0, 1) \tag{15}$$
$$\sigma^{\text{dev}} \sim \text{Exponential}(1) \tag{16}$$
$$\beta^{\text{dev}} \sim \text{Normal}(0, 1) \tag{17}$$
$$\beta^{\text{direct}} \sim \text{Normal}(0, 1) \tag{18}$$

Sibling effects are captured by:

$$S_c \sim \text{Bernoulli}(p^{\text{sibs}}) \tag{19}$$
$$p^{\text{sibs}} \sim \text{Uniform}(0, 1) \tag{20}$$
$$\beta^{\text{OCH}} \sim \text{Normal}(0, 1) \tag{21}$$
$$\beta^{\text{ADU}} \sim \text{Normal}(0, 1) \tag{22}$$

Notation:

- $v_{k,s}^{\text{recs}}$: vocalization count for speaker s in recording k
- $\mu_{c,s}^{\text{child}}$: expected vocalization rate for speaker s for child c
- $\alpha_{\text{child}}^{s}$: variance parameter for speaker s at child level
- $\alpha_{\text{pop},d}^{s}$: variance parameter for speaker s at population level
- μ_s^{pop}: population-level average for speaker s
- α_c^{dev}: child-specific age effect
- β^{dev}, β^{direct}: developmental coefficients
- S_c: indicator for whether child c has siblings

A.1.2 Justification for the assumptions

Our multi-hierarchical model implements several assumptions via equations that relate different variables to one another. Although the speech behavior model's shape is not per se a contribution of the present paper, we informed it on previous research as described in Table 2.

**Assumption**	**How the assumption is justified**	**Relevant equations**
Individual variation in voc count	Much previous research suggests there is individual variation in how voluble families are (e.g. Bergelson et al., 2023)	9 and 10
Sibs → OCH	Having siblings may affect the number of "other child" vocalizations found because there are more children around. Note that the distribution (eq. 21) includes the null as a possible outcome.	9
Sibs → ADU	Having siblings may affect how much adults speak around the key child (Laing & Bergelson). Note that the distribution (eq. 22) includes the null as a possible outcome.	10
No individual variation in how much newborns vocalize	Although individuals may vary already at birth in their language skills, according to data like that in Bergelson et al. (2023) vocalization rates are incredibly low even at around 3 months relative to vocalization rates later in development, such that variation at birth is likely negligible, so we can simplify our model by not including individual variation in children's vocalization rates at age zero.	(Implicit in eq. 8, since the relevant terms are multiplied by age)
Individual variation in how much children vocalize increases with age	Bergelson et al. (2023) and much other work supposes there is relevant individual variation in children's vocalization rates, with increases in this divergence with age.	8
Adults' voc → how much children vocalize	Bergelson et al. (2023) and much other work supposes there are long-term effects of adults' vocalization quantities on children's vocalization rates.	8

Table 2: Model assumptions, justifications, and relevant equations.

Our model of speech behavior allows us to look at a number of downstream effects of confusion errors, while limiting complexity. Future work relying on more data could consider increasing the complexity of the speech model by adding assumptions like the following: (1) Children's age may affect the quantity of vocalizations of others (e.g., perhaps people vocalize more around older children). (2) Number of siblings and the siblings' ages could affect the quantity of vocalization of other children (and perhaps that

of adults).

A.1.3 Fitting the model on human annotations alone

Recordings for which human annotations are available are only partially annotated (typically 30 minutes of audio is annotated, out of many hours). We therefore make the assumption that the relationship between the manual vocalization counts ($\boldsymbol{n_k}^{\text{human}} = (n_{k,CHI}^{\text{human}}, n_{k,OCH}^{\text{human}}, n_{k,FEM}^{\text{human}}, n_{k,MAL}^{\text{human}})$) and the unobserved vocalization counts for the whole recording ($\boldsymbol{v_k}$) is:

$$\boldsymbol{n_k}^{\text{human}} \sim \text{Poisson}\left(\frac{\tau_{\text{annotated}}}{\tau_{\text{rec}}} \cdot \boldsymbol{v_k}\right) \tag{23}$$

Where $\tau_{\text{annotated}}$ is the duration of the audio that was hand-annotated. This makes the drastic (and false) assumption that the vocalization rate is constant throughout the recordings, leading to overconfident credible intervals. Thus, in reality, human annotations alone are even *less* informative than we report in Figure 8. The benefit of complementing human annotations with automated annotations is thus even larger.

A.1.4 Observation and parameters summary

Level	Observations		Parameters		
	Variable	Dimensions	Variable	Dimensions	Prior
**Recordings**	n^{recs}	1401 × 4	$v^{\text{recs}}_{k,\text{CHI}}$	1401	$\text{Gamma}(\alpha^{\text{child}}, \alpha^{\text{child}}/\mu^{\text{rec}}_k)$
	age	1401	$v^{\text{recs}}_{k,s}$ ($s \neq$CHI)	1401 × 3	$\text{Gamma}(\alpha^{\text{child}}, \alpha^{\text{child}}/\mu_c)$
			λ^k_{ij}	1401 × 4 × 4	$\text{Gamma}(\alpha_{ij}, \alpha_{ij}/\mu_{ij})$
	**Total**	**9807**		**28020**	
**Children**	S_c	217	μ_c	217 × 3	$\text{Gamma}(\alpha^{\text{pop}}_d, \alpha^{\text{pop}}_{S_c}/(\mu^{\text{pop}} \exp(S_c\beta)))$
			α^{dev}_c	217	$\text{Normal}(\alpha^{\text{dev}}, \sigma^{\text{dev}})$
	**Total**	**217**		**868**	
**Calibration**	Clip recording	5226	(λ_{ij})	97 × 4 × 4	$\text{Gamma}(\alpha_{ij}, \alpha_{ij}/\mu_{ij})$
	v^{clips}	5226 × 4			
	n^{clips}	5226 × 4			
	**Total**	**78390**		**1552**	
**Population**			(α_{ij})	4 × 4	$\text{Pareto}(1, 1.5)$
			(μ_{ij})	4 × 4	$\mu_{ii} : \text{Exp}(1)$, $\mu_{i \neq j} : \text{Exp}(10)$
			τ	1	Exponential(1)
			α^{child}	4	$\text{Gamma}(4, 1)$
			α^{pop}	2 × 3	$\text{Gamma}(8, 1)$
			μ	4	$\text{Gamma}(2, 8)$
			$\beta^{\text{sib}}_{\text{OCH}}$	1	$\text{Normal}(0, 1)$
			$\beta^{\text{sib}}_{\text{ADU}}$	1	$\text{Normal}(0, 1)$
			p_{sib}	1	$\text{Uniform}(0, 1)$
			α_{dev}	1	$\text{Normal}(0, 1)$
			σ_{dev}	1	Exponential(1)
			β^{dev}	1	$\text{Normal}(0, 1)$
			β^{direct}	1	$\text{Normal}(0, 1)$
	**Total**	**0**		**54**	

Table 3: Summary of observations and parameters entering the joint model.

A.1.5 Stan parameters

	Chains	Warmup iter.	Sampling iter.	Accept. delta	Max tree-depth	CPU cores	Runtime (h)
**Behavioral model**	4	2,000	2,000	0.95	15	48	0.5-1
**Full model (with calibration)**	1	1,000	1,000	0.95	15	48	10-12

A.2 Validation

A.2.1 Confusion model

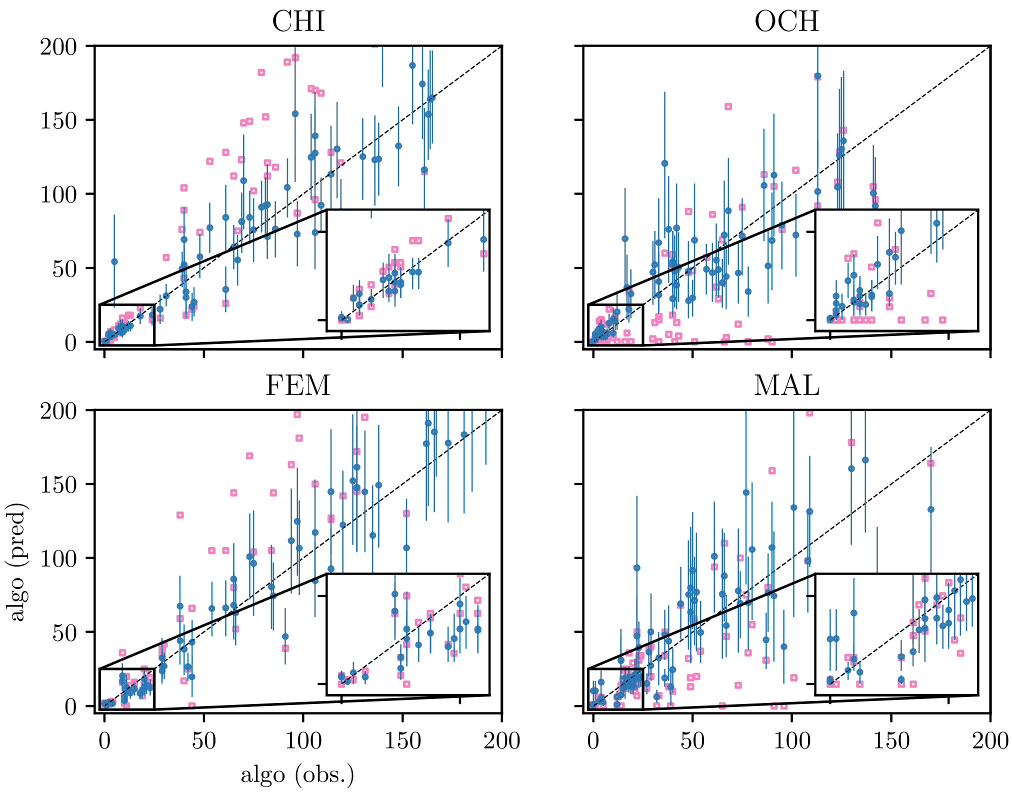

Figure 10: Relationship between the vocalization counts actually derived with VTC and the quantities expected to be derived from VTC given the model of the algorithm behavior and the true vocalization counts. Each blue point represents one of the recordings from the calibration data. The x-axis indicates the amount of vocalizations detected by VTC for each speaker. The y-axis represents the amount expected based on the algorithm behavior (in blue) and the true amount of vocalizations for each speaker (in pink). The error bars indicate 68% probable intervals; most of the uncertainty lies in the variance in the confusion rates across recordings.

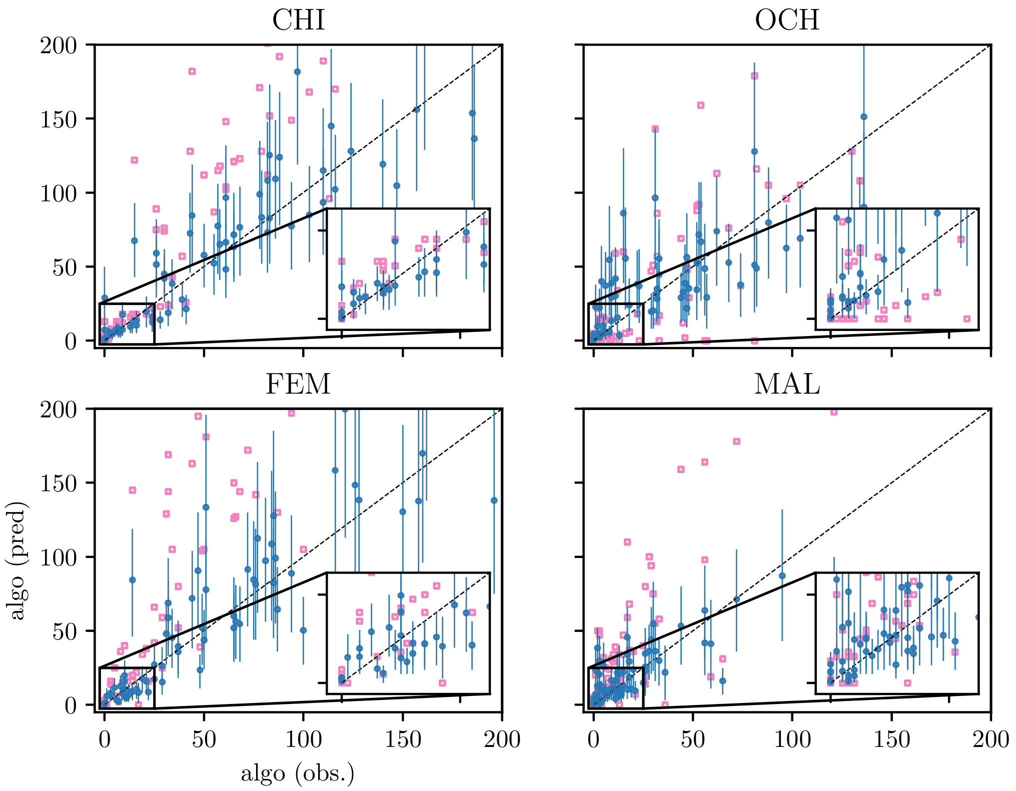

Figure 11: Relationship between the vocalization counts actually derived with LENA™ and the quantities expected to be derived from LENA™ given the model of the algorithm behavior and the true vocalization counts. Each blue point represents one of the recordings from the calibration data. The x-axis indicates the amount of vocalizations detected by LENA™ for each speaker. The y-axis represents the amount expected based on the algorithm behavior (in blue) and the true amount of vocalizations for each speaker (in pink). The error bars indicate 68% probable intervals; most of the uncertainty lies in the variance in the confusion rates across recordings.

A.2.2 Confusion model (validation via simulations)

To further validate our approach, we apply the model to simulated human and algorithmic annotations comparable in size to our calibration dataset. We draw vocalizations by assuming a Poisson process. Vocalization durations are drawn uniformly between 1s and 2s. Confusion rates across simulated recordings are drawn from a Beta distribution with mean μ_{ij} and shrinkage parameter $\eta = 50$. Simulations show that our approach is able to identify the correct confusion rates as long as speech density remains not too high (Figure 12).

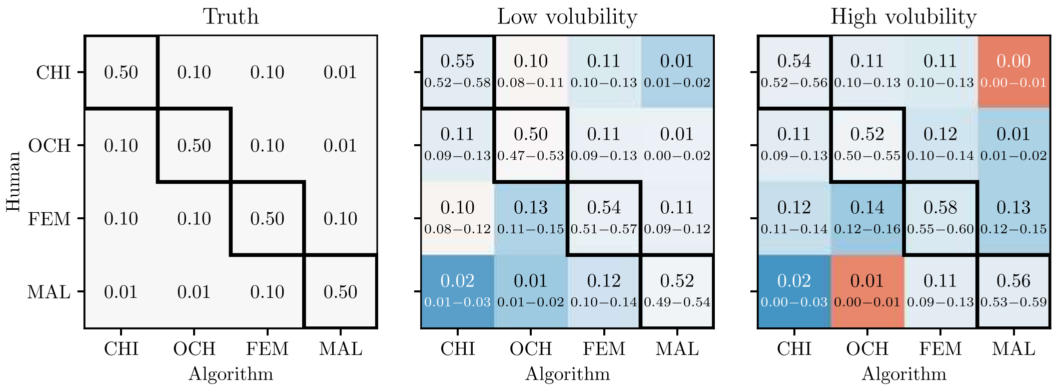

Figure 12: True confusion (μ_{ij}) compared to the confusion matrices recovered by the model from simulated data, under normal and high volubility. Colors indicate deviations from the true values (blue indicates overestimates, and red indicates underestimates). High volubility can lead to overestimating (μ_{ij}).

A.2.3 Bayesian calibration

VTC/LENA comparison before and after calibration

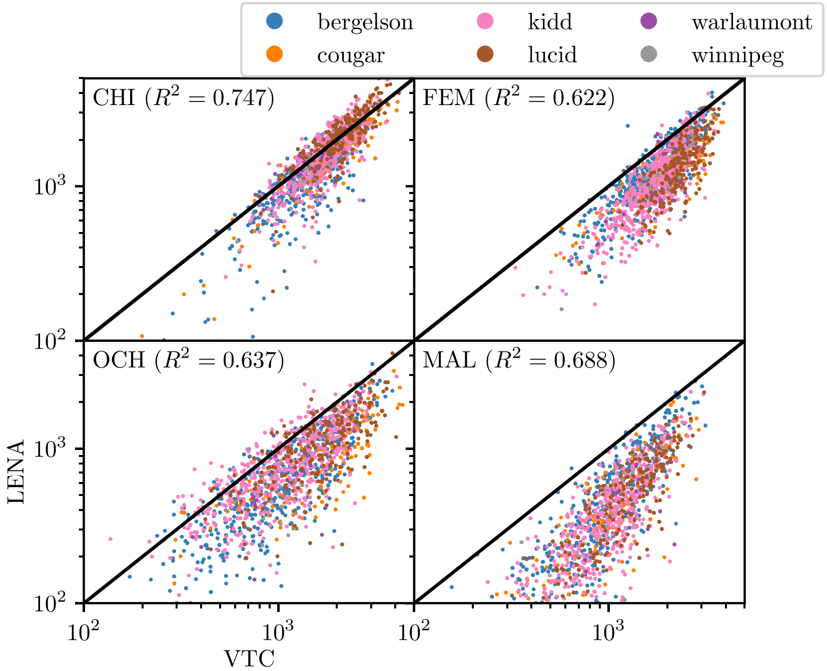

Figure 13: Comparison of vocalization counts derived with VTC (x-axis) and LENA™ (y-axis) per speaker and per recording, prior to any calibration.

	CHI	OCH	FEM	MAL
R^2				
Before calibration	0.747 [0.72, 0.77]	0.637 [0.61, 0.67]	0.622 [0.59, 0.65]	0.688 [0.66, 0.71]
After calibration	0.743 [0.72, 0.77]	**0.713*** [0.69, 0.74]	**0.733*** [0.71, 0.76]	**0.748*** [0.72, 0.77]

Table 4: Correlation (R^2) between LENA and VTC vocalization counts measured for each speaker, with and without calibration. Brackets indicate 95% confidence intervals. Best values are shown in bold, when before/after differences are significant (∗).

Automated versus manual estimates

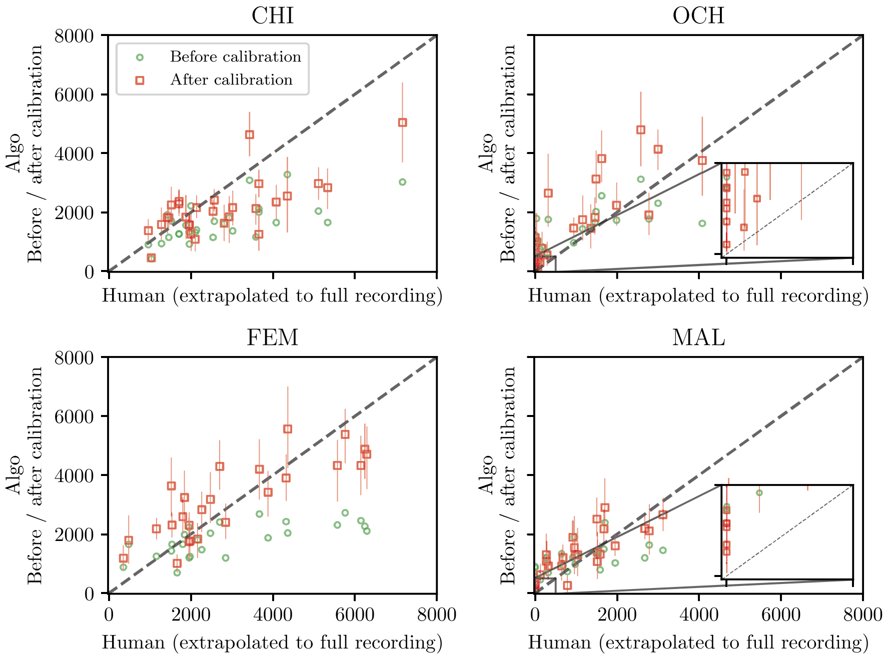

Figure 14: Comparison of manual estimates, uncalibrated VTC estimates, and calibrated VTC posterior estimates of total vocalization counts in recordings partially covered by human annotations. Prior to calibration, biases are manifest (CHI, FEM and MAL are almost systematically underestimated, and OCH is almost systematically overestimated).

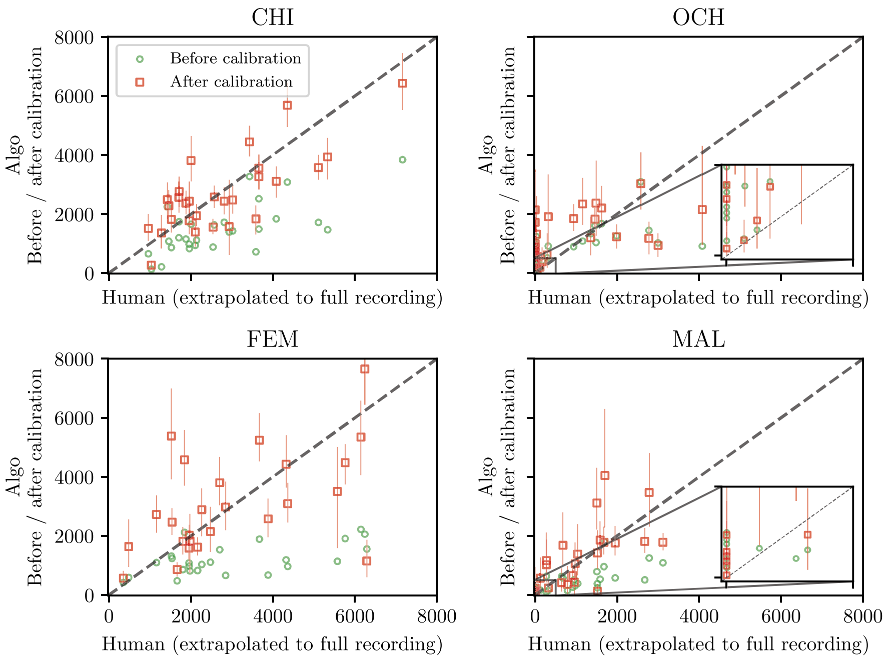

Figure 15: Comparison of manual estimates, uncalibrated LENA estimates, and calibrated LENA posterior estimates of total vocalization counts in recordings partially covered by human annotations.

N	CHI ($N = 28$)	OCH ($N = 28$)	FEM ($N = 28$)	MAL ($N = 28$)
R^2				
Before (algo)	0.432 [0.14, 0.68]	0.523 [0.23, 0.75]	0.667 [0.41, 0.83]	0.411 [0.12, 0.67]
After (calibration)	0.510 [0.22, 0.74]	0.708 [0.47, 0.85]	**0.859*** [0.72, 0.93]	**0.698*** [0.45, 0.85]
**ICC**				
Before (algo)	0.213 [0.00, 0.54]	0.587 [0.28, 0.78]	0.152 [0.00, 0.49]	0.481 [0.14, 0.72]
After (calibration)	**0.567*** [0.26, 0.77]	0.732 [0.50, 0.87]	**0.849*** [0.70, 0.93]	**0.795*** [0.61, 0.90]

VTC

N	CHI ($N = 28$)	OCH ($N = 28$)	FEM ($N = 28$)	MAL ($N = 28$)
R^2				
Before (algo)	0.458 [0.17, 0.70]	0.417 [0.13, 0.67]	0.629 [0.36, 0.81]	0.391 [0.11, 0.66]
After (calibration)	0.604 [0.32, 0.79]	0.288 [0.04, 0.57]	0.667 [0.41, 0.83]	0.440 [0.15, 0.69]
**ICC**				
Before (algo)	0.252 [0.00, 0.57]	0.568 [0.26, 0.77]	0.023 [0.00, 0.39]	0.233 [0.00, 0.55]
After (calibration)	**0.779*** [0.58, 0.89]	0.472 [0.13, 0.71]	**0.811*** [0.64, 0.91]	**0.672*** [0.41, 0.83]

LENA

Table 5: Agreement between algorithmic vocalization counts (before/after calibration) and manual vocalization counts extrapolated to whole recordings. Brackets indicate 95% confidence intervals. Best-values are shown in bold, when differences are statistically significant ($*$).

A.2.4 Bayesian calibration (validation via simulations)

We provide an additional layer of validation of the calibration approach by running the procedure on synthetic vocalization data simulated under fixed values of the parameters of interest. We set plausible values for the effects that are expected to be large in real-life ($\alpha^{\text{dev}} = 0.5$, $\sigma^{\text{dev}} = 0.1$, $\beta^{\text{OCH}} = -1$) and zero-values for less trivial effects ($\beta^{\text{ADU}} = \beta^{\text{direct}} = \beta^{\text{dev}} = 0$). We set the priors such that $\mu^{\text{FEM}}/(\mu^{\text{FEM}} + \mu^{\text{MAL}}) = 0.8$. We simulate vocalization counts for 1000 observations (200 children with 5 observations each).

For each observation, we simulate automated vocalization counts using the procedure described in Section §2.2. We estimate the model parameters using three different inputs: a) the true synthetic vocalization counts; b) the simulated automated vocalization counts, without calibration; and c) the simulated automated vocalization counts, with calibration. The results are shown in Figure 17. This confirms that algorithmic estimates can be strongly biased, and that calibration generally reduces the tension between the ground truth and the posterior estimates.

N	CHI ($N=1000$)	OCH ($N=1000$)	FEM ($N=1000$)	MAL ($N=1000$)
R^2				
Before (algo)	0.608 [0.57, 0.64]	0.172 [0.13, 0.22]	0.417 [0.37, 0.46]	0.060 [0.03, 0.09]
After (calibration)	**0.723*** [0.69, 0.75]	**0.565*** [0.52, 0.60]	**0.578*** [0.54, 0.62]	**0.161*** [0.12, 0.20]
**Relative error**				
Before (algo)	0.847 [0.81, 0.89]	1.119 [1.05, 1.19]	1.297 [1.24, 1.35]	2.108 [1.93, 2.29]
After (calibration)	**0.540*** [0.51, 0.57]	**0.685*** [0.65, 0.72]	**0.653*** [0.62, 0.69]	**0.979*** [0.94, 1.02]

VTC

N	CHI ($N=1000$)	OCH ($N=1000$)	FEM ($N=1000$)	MAL ($N=1000$)
R^2				
Before (algo)	0.429 [0.38, 0.48]	0.175 [0.13, 0.22]	0.298 [0.25, 0.35]	0.116 [0.08, 0.16]
After (calibration)	**0.601*** [0.56, 0.64]	**0.563*** [0.52, 0.60]	**0.447*** [0.40, 0.49]	**0.185*** [0.14, 0.23]
**Relative error**				
Before (algo)	1.081 [1.03, 1.14]	1.120 [1.07, 1.17]	1.708 [1.64, 1.78]	1.793 [1.72, 1.87]
After (calibration)	**0.640*** [0.60, 0.68]	**0.670*** [0.64, 0.71]	**0.760*** [0.73, 0.80]	**0.962*** [0.92, 1.00]

LENA

Table 6: Accuracy of automated estimates, evaluated in terms of R^2 and relative error ($\frac{\text{RMSE}}{\sigma_{\text{truth}}}$), before calibration (using the simulated algorithm counts) and after calibration (using $\mathbb{E}(v_{ik})$, the posterior expectancy value of the true vocalization counts). Brackets indicate 95% confidence intervals. Best values are shown in bold, when before/after differences are significant ($*$). Calibration significantly improves accuracy in simulated data.

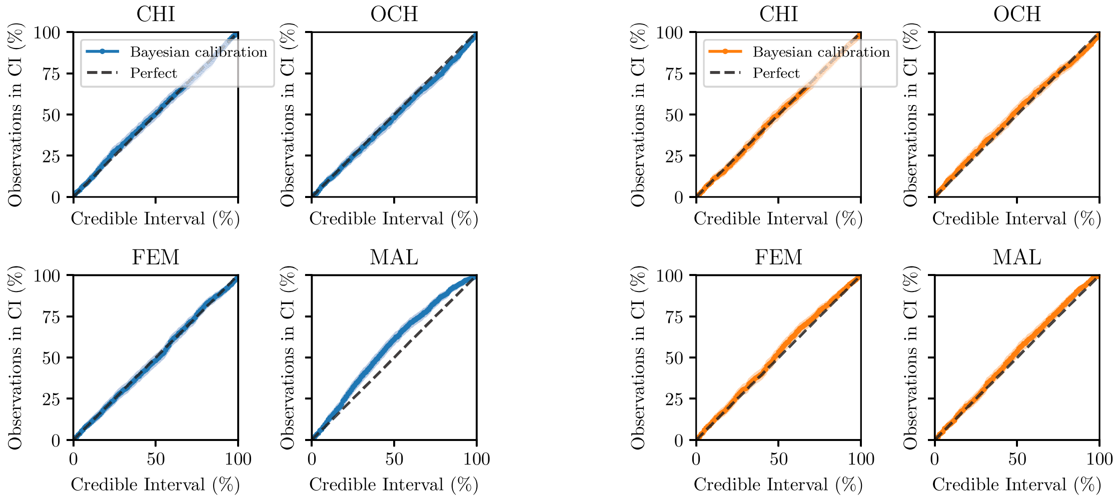

(a) VTC (b) LENA

Figure 16: Calibration of posterior credible intervals for vocalization counts by speaker. Each point shows the proportion of observations falling within credible intervals of a given nominal level (x-axis). Perfect calibration (diagonal line) occurs when, e.g., 90% credible intervals contain the true value 90% of the time.

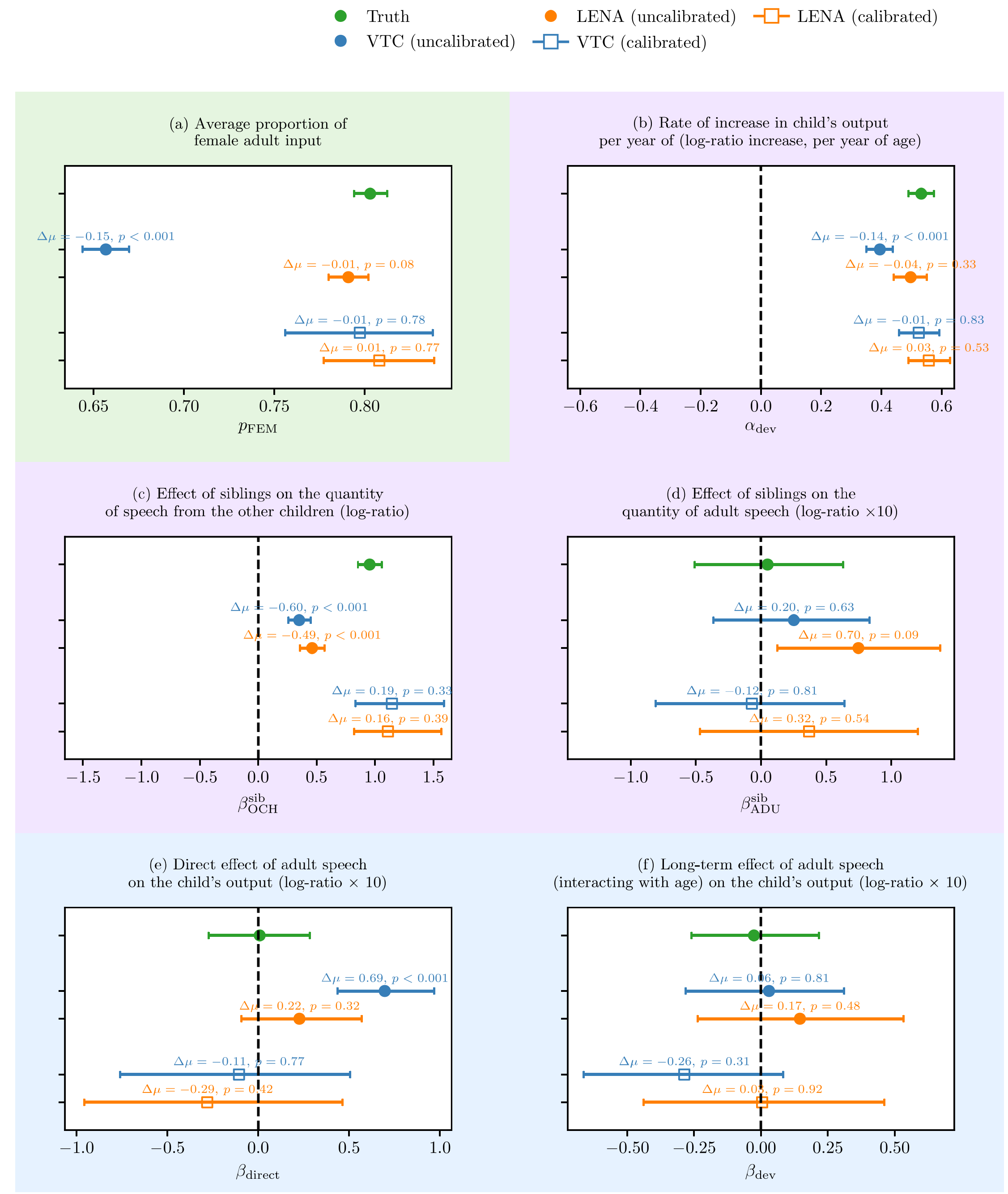

Figure 17: Performance of the calibration procedure on synthetic data. "Truth" values represent the posterior estimates obtained with the synthetic ground truth. $\Delta\mu$ is the difference between automated estimates and truth estimates. The p-values p measure the significance of the difference using a two-tailed permutation test (higher is better). Unlike uncalibrated estimates, calibrated estimates never appear in significant tension with the truth estimates. In fact, calibration almost always reduces the tension (except for β^{dev} with VTC).

A.2.5 Results

		Manual annotations	Automated annotations			
			Prior to calibration		After calibration	
			VTC	LENA	VTC	LENA
**Speech quantity**	Female adult proportion	0.76<br>[0.64, 0.84]	0.64<br>[0.63, 0.65]	0.73<br>[0.71, 0.75]	0.74<br>[0.70, 0.77]	0.78<br>[0.74, 0.82]
**Effect of independent variables on speech quantities**	Age → output	0.18<br>[−0.19, 0.53]	**0.30**<br>**[0.26, 0.35]**	**0.42**<br>**[0.36, 0.48]**	**0.43**<br>**[0.37, 0.49]**	**0.51**<br>**[0.43, 0.58]**
	Siblings → input from children	**1.34**<br>**[0.28, 2.29]**	**0.58**<br>**[0.48, 0.68]**	**0.64**<br>**[0.54, 0.75]**	**1.14**<br>**[0.93, 1.37]**	**1.06**<br>**[0.86, 1.24]**
	Siblings → adult input	−0.78<br>[−2.61, 1.04]	−0.48<br>[−1.06, 0.11]	**−1.39**<br>**[−2.12, −0.62]**	**−1.84**<br>**[−2.73, −0.96]**	**−1.90**<br>**[−2.91, −0.85]**
**Associations between speech quantities**	Input → output (direct)	−0.18<br>[−1.71, 1.36]	**0.73**<br>**[0.57, 0.89]**	**1.16**<br>**[0.91, 1.41]**	**0.71**<br>**[0.41, 1.02]**	**1.19**<br>**[0.80, 1.55]**
	Input → output (long-term)	−0.07<br>[−1.78, 1.62]	**0.50**<br>**[0.26, 0.75]**	0.24<br>[−0.07, 0.54]	**0.54**<br>**[0.20, 0.93]**	0.27<br>[−0.13, 0.70]

Table 7: Comparison of regression estimates from manual and automated annotations, including their posterior 95% credible intervals. Significant effects appear in bold characters.

A.2.6 Alternative approach (direct comparison)

An undesirable feature of the approach based on vocalization counts in 15s clips is that reliance on an arbitrary window-size. Shorter windows introduce boundary effects; longer windows decrease the informativeness of each individual clip, challenging the ability of the model to learn the distribution of confusion rates.

We considered an alternative strategy, based on an direct comparison of the vocalizations retrieved by the algorithms and the human annotators. In this strategy, an algorithmic vocalization attributed to a speaker i is considered a true positive iif it has a non-zero intersection with a real vocalization from the same speaker. It is considered a false positive iif it has no intersection with actual vocalizations from the correct speaker (i), but intersects with vocalizations from a single other speaker $j \neq i$. For each recording k with human annotations, we thus directly derive an estimate of n_{kij}, the amount of vocalizations attributed to j as a result of vocalizations from i. This approach, however, necessarily underestimates confusion rates; in particular, it fails to capture vocalizations misidentified every time a detected vocalization intersects with actual vocalization from two speakers. Figure 18 confirms that this yields lower confusion rates than the 15s clips method.

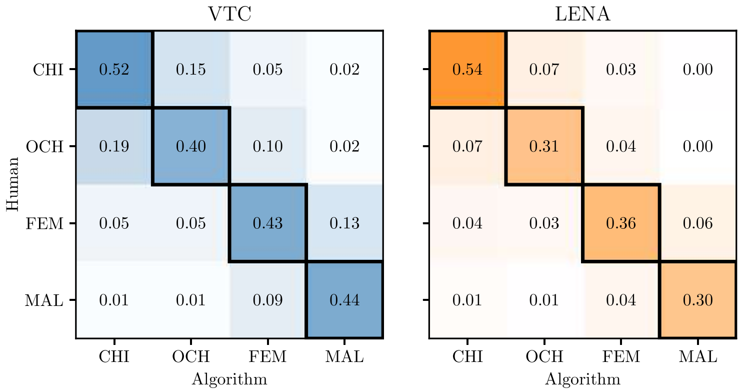

Figure 18: Average confusion rates of VTC and LENA™ estimated within our model. Rows indicate the true speaker, and columns indicate the speaker class attributed by the algorithm. Diagonal elements represent the true positive rate for each speaker. Non-diagonal elements represent the distributions of the rates of false positives.

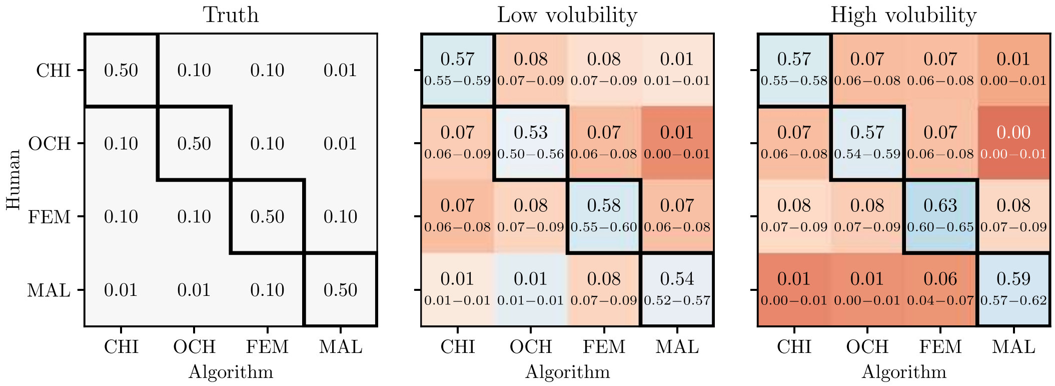

Figure 19: True confusion (μ_{ij}) compared to the confusion matrices recovered by the alternative inference strategy from simulated data, under normal and high volubility. The matrix to the left represents the true values. Colors indicate deviations from the true values (blue indicates overestimates, and red indicates underestimates). The alternative strategy generally underestimates misclassification errors and overestimates true positives.

A.2.7 Downstream comparison of the two calibration strategies

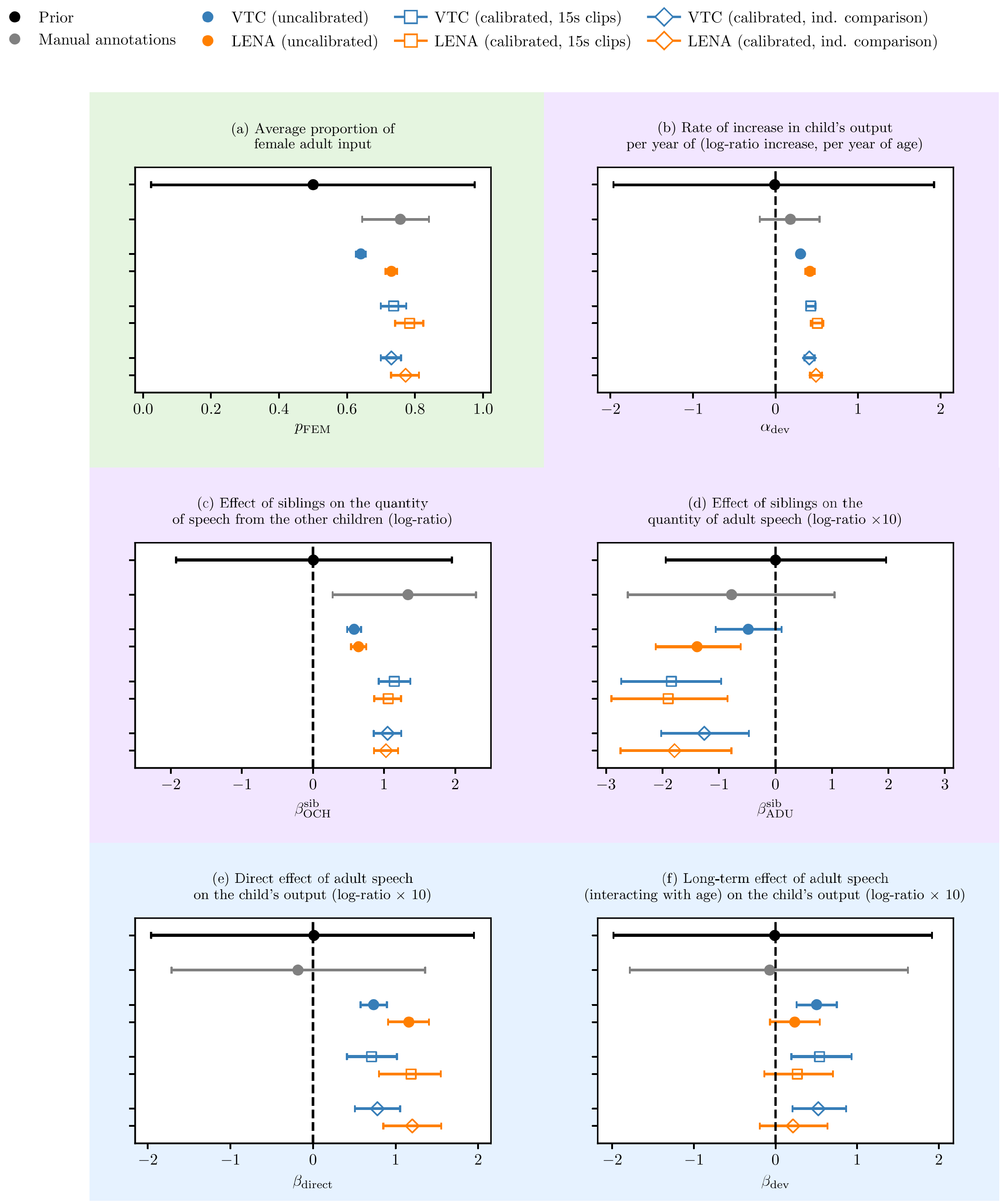

Figure 20: Comparison of effects' sizes derived with manual annotations alone (in gray) and automated annotations (in colors), without any calibration and with calibration. The prior distribution ($\mathcal{N}(0,1)$ or $\mathcal{U}(0,1)$, depending on the variable support) is shown in black for purposes of comparison. We distinguish three types of measurements: direct measurements of speech quantities (a); measurements of the effect of an independent variable on speech quantity (b, c, d); and measurements of the effect of a quantity of speech on another quantity of speech (e, f).

A.3 Effect of the child's age and environment on confusion rates

Figures 7a and 7b collapse across all children and corpora. In reality, the confusion matrix might depend on a number of factors, in which case collapsing across them is inappropriate. One of them is the child's age, which could affect the ability of the algorithm to correctly detect and classify children's vocalization. If that were the case, this could potentially undermine the ability of inferring the effect of age on the child's speech production. The effect of age on the algorithm performance on the vocalizations of the key child is shown for different age groups in Figure 21. There is some evidence that the detection rate for children increases after two years of age.

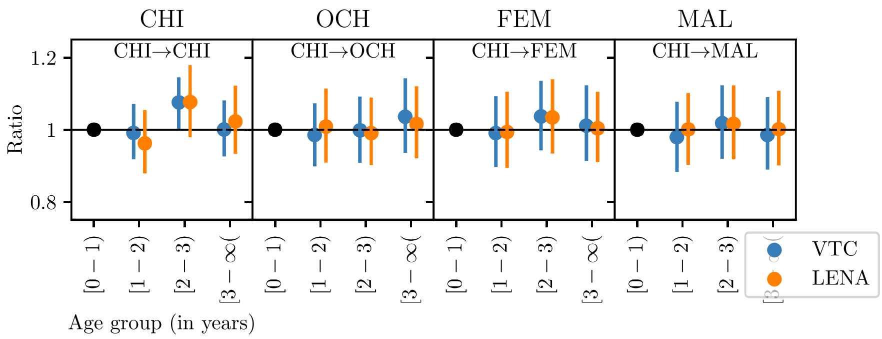

Figure 21: Effect of child age on the confusion rates $\lambda_{\mathrm{CHI},i}$ (CHI→ $i \in$ {CHI,OCH,FEM,MAL}). The mean confusion rate for each age bin is compared the mean confusion rates for children between zero and one year of age (a ratio of one implies no difference). For VTC, variations larger than ∼10% in the mean true positive rates are excluded.

Besides child age, it is also conceivable that confusion rates depend on environmental factors (e.g. time spend outside, exposure to noise, etc.) and vary across languages. We therefore sought to compare the confusion rates for corpora drawing from urban English-speaking populations with confusion rates estimated from recordings of rural and non-English speaking populations. To this end, we drew from corpora that sampled "rural" populations in Papua New Guinea (Cristia & Casillas, 2020), the Solomon Islands (Cassar, Cristia, Grosjean, & Walker, 2025), Vanuatu (Cristia et al., 2023), and Bolivia (Scaff, Casillas, Stieglitz, & Cristia, 2023). Some of these recordings were done with devices other than LENA™. Moreover, all of these languages are considered under-resourced, which may mean that algorithms built on cumulative knowledge in the speech technology literature may be particularly ill-suited to them. In any case, English was seldom if ever spoken in these recordings, which makes them mismatch in training set with LENA™ (which was trained on North American English audio). As a result, our comparison conflates across many dimensions, all of which predict poorer algorithm performance in the "rural" recordings. Thus, while this comparison does not allow to isolate the effect of the environment – urban vs rural –, language, or recording device (which we

cannot do due to limited sample size), it gives an idea about whether any of these factors could alter classification errors.

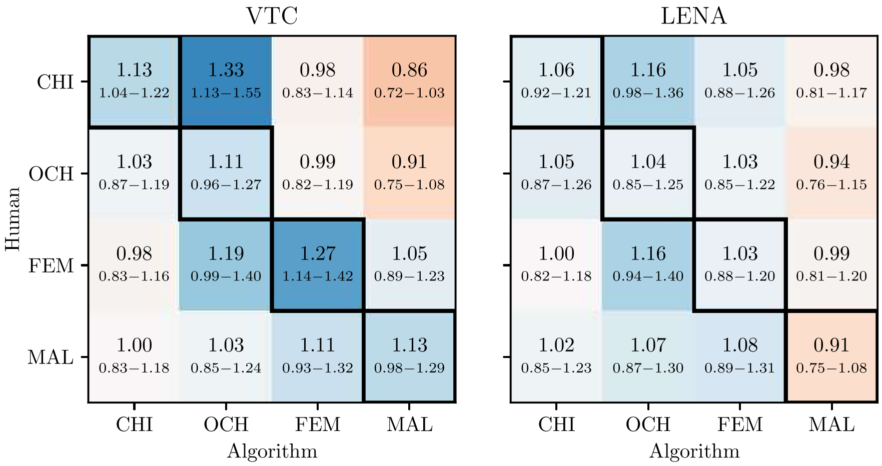

Figure 22: Rural/urban confusion rates for VTC (left) and LENA^{TM} (right). The latter include non-English speaking, non-WEIRD populations and recorders other than LENA^{TM}. Values greater than one (blue cells) signal higher confusion rates among rural corpora than among urban corpora. 95% credible intervals are indicated underneath each value.

Figure 22 finds modest differences, most of them being statistically non-significant.

A.4 The potential of classifiers' confidence scores as covariates

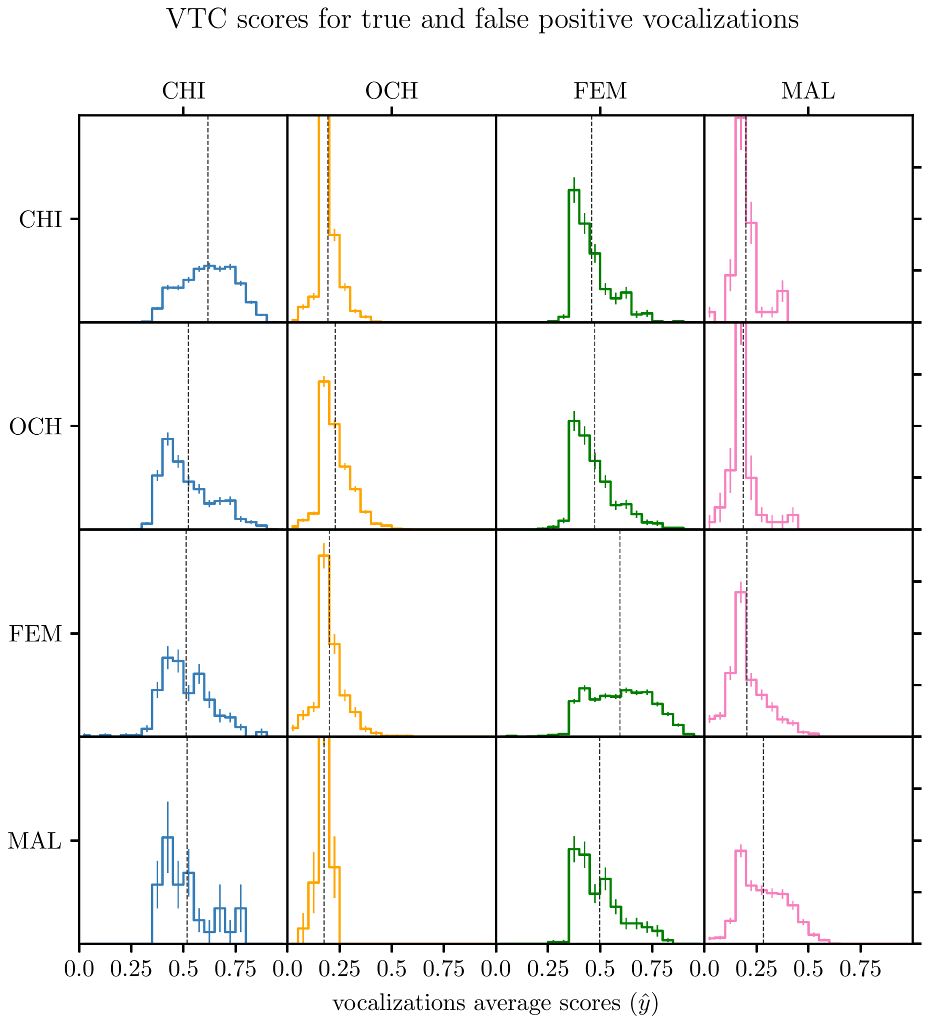

Figure 23: Distribution of the confidence score of VTC for each vocalization given the detected speaker type (in columns) and the true speaker (in rows). Dashed vertical lines indicate the mean of the distribution. Vocalizations for which the speaker is correctly identified exhibit higher confidence scores, which suggests these scores could be used as informative covariates in a calibration approach.

A.5 Inter-rater agreement

Speaker Clips	Human–Human ($N = 61$)	Human–VTC ($N = 61$)	Human–LENA ($N = 61$)	LENA–VTC ($N = 61$)
Child	0.89 [0.82, 0.93]	0.39 [0.16, 0.58]	0.63 [0.46, 0.76]	0.40 [0.16, 0.59]
Other Child	0.72 [0.57, 0.82]	0.49 [0.28, 0.66]	0.45 [0.23, 0.63]	0.64 [0.46, 0.77]
Female Adult	0.80 [0.69, 0.88]	0.43 [0.20, 0.61]	0.15 [0.00, 0.38]	0.50 [0.29, 0.67]
Male Adult	0.82 [0.72, 0.89]	0.54 [0.34, 0.70]	0.40 [0.17, 0.59]	0.41 [0.18, 0.60]

Table 8: Intraclass Correlation Coefficient (ICC) measures of inter-rater agreement, based on vocalization counts in $N = 61\times$ 15s clips annotated by two human annotators.

A.6 VTC and LENA™ produce inconsistent correlation estimates

While revealing of classification errors, correlations between speech quantities measured in short clips bear little importance in themselves. Users are often more interested in correlations between vocalization counts aggregated at the level of whole recordings or the level of each child – those are shown in Figures 24 and 25 respectively. These correlations exhibit similar issues, with VTC and LENA™ reporting inconsistent correlations – particularly for those with vocalizations attributed to other children. This further suggests that the estimation of such correlations is affected by classification errors.

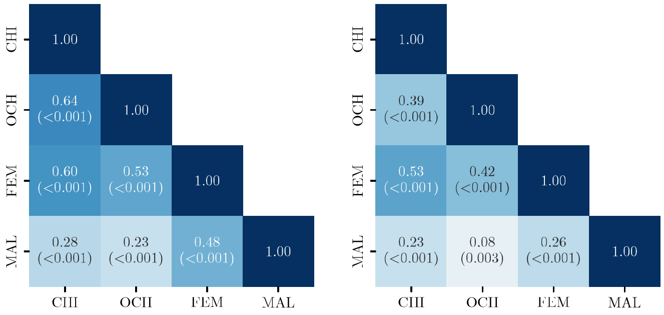

(a) VTC (b) LENA

Figure 24: Correlation between the quantity of vocalizations attributed to each speaker across recordings. The correlation matrix is extracted using a hierarchical multivariate log-normal model described in Section §A.6.1. Estimates from manual annotations are not included due to a lack of data at the full-recording level.

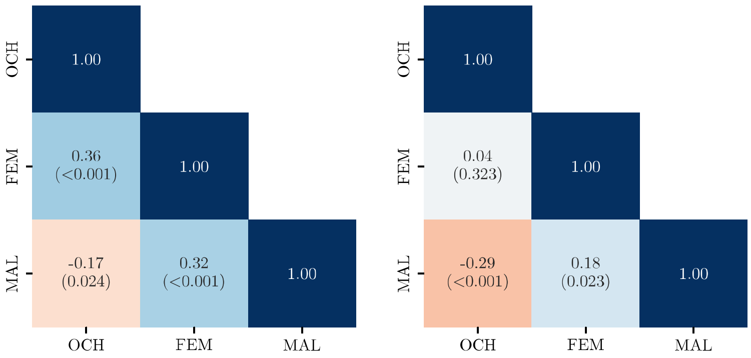

(a) VTC (b) LENA

Figure 25: Correlation between the quantity of vocalizations attributed to each speaker across children. "CHI" is omitted since it varies significantly with age. The correlation matrix is extracted using a hierarchical multivariate log-normal model described in Section §A.6.1.

A.6.1 Surrogate models for correlation estimates

In order to evaluate recording-level and child-level correlations between speakers, we use surrogate models as alternatives to the above model.

Recording-level correlations between speakers In this model for each recording k of child c, the vocalization counts are now modeled using a multivariate log-normal distribution:

$$\log \mathbf{v}_k^{\text{recs}} \sim \text{MVN}\left(\boldsymbol{\mu}_k - \frac{\text{diag}(\Sigma)}{2}, \Sigma\right) \tag{24}$$

where $\mathbf{v}_k^{\text{recs}} = (v_{k,\text{CHI}}^{\text{recs}}, v_{k,\text{OCH}}^{\text{recs}}, v_{k,\text{FEM}}^{\text{recs}}, v_{k,\text{MAL}}^{\text{recs}})$ is the vector of vocalization counts for all speaker types.

The mean vector $\boldsymbol{\mu}_k$ is defined as:

$$\mu_{k,\text{CHI}} = \log(\mu_{\text{CHI}}^{\text{pop}}) + \chi_k \tag{25}$$

$$\mu_{k,s} = \log(\mu_{c,s}^{\text{child}}), \quad s \in \{\text{OCH}, \text{FEM}, \text{MAL}\} \tag{26}$$

The child's developmental effect χ_k is:

$$\chi_k = \alpha_c^{\text{dev}} \cdot \frac{\text{age}_k}{12} + \beta^{\text{dev}} \cdot \frac{\text{age}_k}{12} \cdot \frac{\mu_{c,\text{ADU}}^{\text{child}} - \mu_{\text{ADU}}}{\sigma_{\text{ADU}}} \tag{27}$$

Note that this formulation removes the direct effect term (β^{direct}) that was present in the original model and introduces a covariance structure Σ between the different speaker types. The term $-\text{diag}(\Sigma)/2$ ensures the expected values after exponentiation match the

desired means.

The covariance matrix Σ is parameterized through its Cholesky decomposition L_Σ such that $\Sigma = L_\Sigma L_\Sigma^T$.

All other aspects of the model (child-level parameters, population-level parameters, developmental effects, and sibling effects) remain unchanged from the original specification.

Child-level correlations between speakers In a third model aimed at measuring correlations between speakers at the child-level, the child-level parameters are assumed to follow a multivariate log-normal distribution:

$$\log(\boldsymbol{\mu}_c^{\text{child}}) \sim \text{MVN}\left(\log(\boldsymbol{\mu}_{2:n}^{\text{pop}}) - \frac{\text{diag}(\Sigma_{\text{child}})}{2}, \Sigma_{\text{child}}\right) \tag{28}$$

where $\boldsymbol{\mu}_c^{\text{child}} = (\mu_{c,\text{OCH}}^{\text{child}}, \mu_{c,\text{FEM}}^{\text{child}}, \mu_{c,\text{MAL}}^{\text{child}})$ and $\boldsymbol{\mu}_{2:n}^{\text{pop}} = (\mu_{\text{OCH}}^{\text{pop}}, \mu_{\text{FEM}}^{\text{pop}}, \mu_{\text{MAL}}^{\text{pop}})$.

The parameters for defining the adult speech influence are now:

$$\mu_{\text{ADU}} = \mu_{\text{FEM}}^{\text{pop}} + \mu_{\text{MAL}}^{\text{pop}} \tag{29}$$

$$\sigma_{\text{ADU}} = \sqrt{(\exp(\Sigma_{\text{FEM,FEM}}^{\text{child}}) - 1)(\mu_{\text{FEM}}^{\text{pop}})^2 + (\exp(\Sigma_{\text{MAL,MAL}}^{\text{child}}) - 1)(\mu_{\text{MAL}}^{\text{pop}})^2} \tag{30}$$

All other model components (developmental effects, population-level parameters) remain unchanged from the first model.

The key difference in this model is that it captures correlations between different speaker types at the child level through the multivariate log-normal distribution, while maintaining the original Gamma distributions for the recording-level observations.

Level	Observations		Parameters		
	**Variable**	**Dimensions**	**Variable**	**Dimensions**	**Prior**
**Recordings**	n^{recs}	1401×4			
	age	1401			
	**Total**	**9807**		**0**	
**Children**	S_c	217	μ_c	217×3	$\text{Gamma}(\alpha_d^{\text{pop}}, \alpha_{S_c}^{\text{pop}}/(\mu^{\text{pop}} \exp(S_c\beta)))$
			α_c^{dev}	217	$\text{Normal}(\alpha^{\text{dev}}, \sigma^{\text{dev}})$
	**Total**	**217**		**868**	
**Population**			α^{child}	4	$\text{Gamma}(4, 1)$
			α^{pop}	2×3	$\text{Gamma}(8, 1)$
			μ	4	$\text{Gamma}(2, 8)$
			L_Ω	4×4	LKJCholesky(1)
			L_σ	4	$\text{Gamma}(2, 4)$
			$\beta_{\text{OCH}}^{\text{sib}}$	1	$\text{Normal}(0, 1)$
			$\beta_{\text{ADU}}^{\text{sib}}$	1	$\text{Normal}(0, 1)$
			p_{sib}	1	$\text{Uniform}(0, 1)$
			α_{dev}	1	$\text{Normal}(0, 1)$
			σ_{dev}	1	Exponential(1)
			β^{dev}	1	$\text{Normal}(0, 1)$
			β^{direct}	1	$\text{Normal}(0, 1)$
	**Total**	**0**		**41**	

Table 9: Summary of observations and parameters entering the multivariate uncalibrated models.

A.7 Aggregating across multiple algorithms

Our approach could be adapted to aggregate annotations from multiple algorithms. We decided to leave this to future work for both conceptual and practical reasons. On the conceptual side, the present work draws from VTC and LENA™, which mostly differ in overall performance and recall-precision trade-off. We expect gains in such a case to be minimal, in contrast to combining two algorithms that have complementary abilities; e.g., if algorithm A was better at telling apart male from female, and algorithm B was better at telling apart key child from other children. On the practical side, the model described below failed to converge within the time limits imposed by our cluster (48h per chain), suggesting its practical applicability is limited under traditional Hamiltonian Monte Carlo.

Aggregating across two algorithms, the likelihood factorizes such that

$$\log P(\text{algo}(A), \text{algo}(B) \mid \text{truth}) = \log P(\text{algo}(A) \mid \text{truth}) + \log P(\text{algo}(B) \mid \text{truth}).$$

This means that in Stan, the likelihood computation can be decomposed accordingly:

```
model {
    // ...
    // inverse confusion model
    target += reduce_sum(
```

```
        inverse_model_lpdf, actual_confusion_algo1, 1,
        n_recs, n_classes, recs_duration,
        vocs_algo1, truth_vocs, tau1
    );
    target += reduce_sum(
        inverse_model_lpdf, actual_confusion_algo2, 1,
        n_recs, n_classes, recs_duration,
        vocs_algo2, truth_vocs, tau2
    );
    // ...
}
```

Since there can be correlations between the errors made by different algorithms, we modelled these correlations as follows:

```
for (k in 1:n_recs) {
        actual_confusion_rate_algo1[k] = confusion_baseline_algo1[k];
        actual_confusion_rate_algo2[k] = confusion_baseline_algo2[k] .* exp(0.1 *
    ↪ beta_algo1_algo2 .* (actual_confusion_rate_algo1[k]./mus_algo1-1));
}
```

The implementation of this model can be found at https://gin.g-node.org/LAAC-LSCP/speaker-confusion-model/src/main/code/models/dev_combined_simple.stan
